# A PI3Kδ-Foxo1-FasL signaling amplification loop rewires CD4^+^ T cell signaling and differentiation

**DOI:** 10.1084/jem.20252154

**Published:** 2026-02-20

**Authors:** Dominic P. Golec, Pedro H. Gazzinelli-Guimaraes, Daniel Chauss, Kang Yu, Hiroyuki Nagashima, Anthony C. Cruz, Tom Hill, Sundar Ganesan, Jennifer L. Cannons, Jillian K. Perry, Luis Nivelo, Ilin Joshi, Nicolas Pereira, Fabrício Marcus Silva Oliveira, Yufan Zheng, Makheni Jean Pierre, Kirk M. Druey, Justin B. Lack, Eric V. Dang, Thomas B. Nutman, Alejandro V. Villarino, John J. O’Shea, Behdad Afzali, Pamela L. Schwartzberg

**Affiliations:** 1 Laboratory of Immune System Biology, National Institute of Allergy and Infectious Diseases, National Institutes of Health, Bethesda, MD, USA; 2 Department of Immunology, University of Pittsburgh School of Medicine, Pittsburgh, PA, USA; 3 Department of Microbiology, Immunology and Tropical Medicine, School of Medicine and Health Science, George Washington University, Washington, DC, USA; 4 Immunoregulation Section, National Institute of Diabetes and Digestive and Kidney Diseases, National Institutes of Health, Bethesda, MD, USA; 5 Lymphocyte Cell Biology Section, National Institute of Arthritis and Musculoskeletal and Skin Diseases, National Institutes of Health, Bethesda, MD, USA; 6 National Institute of Allergy and Infectious Diseases Collaborative Bioinformatics Resource, National Institute of Allergy and Infectious Diseases, National Institutes of Health, Bethesda, MD, USA; 7 Research Technologies Branch, National Institute of Allergy and Infectious Diseases, National Institutes of Health, Bethesda, MD, USA; 8 Department of Microbiology and Immunology, Miller School of Medicine, University of Miami, Miami, FL, USA; 9 Laboratory of Allergic Diseases, National Institute of Allergy and Infectious Diseases, National Institutes of Health, Bethesda, MD, USA; 10 Laboratory of Host Immunity and Microbiome, National Institute of Allergy and Infectious Diseases, National Institutes of Health, Bethesda, MD, USA; 11 Department of Microbiology and Immunology, Georgetown University, Washington, DC, USA; 12 Ragon Institute of Mass General Hospital, Harvard, and MIT, Cambridge, MA, USA; 13 Laboratory of Parasitic Diseases, National Institute of Allergy and Infectious Diseases, National Institutes of Health, Bethesda, MD, USA

## Abstract

While inputs regulating CD4^+^ T helper (Th) cell differentiation are well defined, the integration of downstream signaling with transcriptional and epigenetic programs that define Th lineage identity remains incompletely resolved. PI3K signaling is a critical regulator of T cell function; activating mutations affecting PI3Kδ result in an immunodeficiency with multiple T cell defects. Using mice expressing activated PI3Kδ, we found aberrant expression of proinflammatory Th1 signature genes under Th2-inducing conditions, both *in vivo* and *in vitro*. This dysregulation was driven by a PI3Kδ-IL-2-Foxo1 signaling amplification loop, fueling Foxo1 inactivation, loss of Th2 lineage restriction, and extensive epigenetic reprogramming. Surprisingly, ablation of *Fasl*, a Foxo1-repressed gene, normalized both Th2 differentiation and TCR signaling. BioID and imaging revealed Fas interactions with TCR signaling components, which were supported by Fas-mediated potentiation of TCR signaling that could occur in the absence of FADD. Our results highlight Fas-FasL signaling as a critical intermediate in phenotypes driven by activated PI3Kδ, thereby linking two key pathways of immune dysregulation.

## Teaser

Golec et al. describe a PI3 Kinase-Foxo1-FasL signaling circuit that promotes amplified signaling and rewires transcriptional and epigenetic programs driving IFN-γ and altered T helper cell differentiation in CD4^+^ T cells from mice expressing an activating mutant of phosphoinositide 3-kinase delta.

## Graphical Abstract

**Figure:**
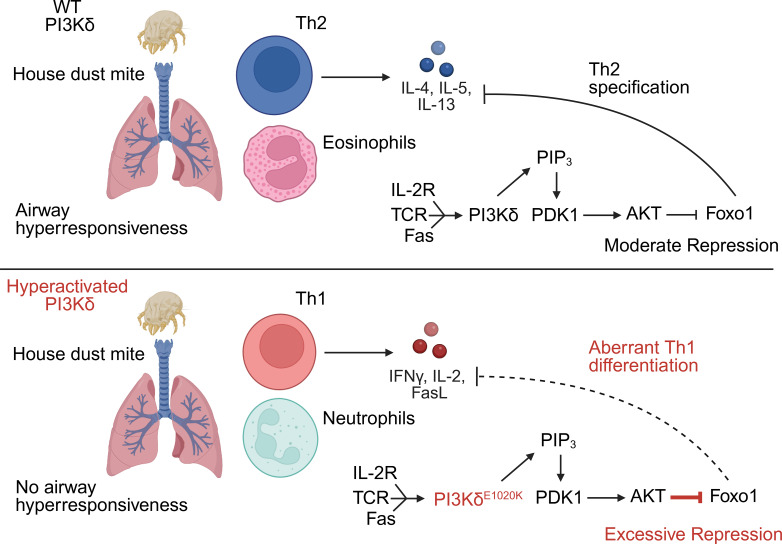

## Introduction

The differentiation of CD4^+^ T cells into distinct types of cytokine-producing T helper (Th) cell populations is critical for orchestrating immune responses to different types of infections and immune challenges (Zhu et al., 2010). Among these, Th1 cells express interferon-gamma (IFNγ), which drives activation of cellular immunity against intracellular pathogens, whereas Th2 cells produce IL-4, IL-5, and IL-13, which promote both hypersensitivity and tissue repair processes. Th cell differentiation requires the translation of extracellular signals from the T cell receptor (TCR) and cytokines into intracellular responses that dictate transcriptional and epigenetic signatures required to establish T cell identity. Nonetheless, how these pathways coalesce to regulate CD4^+^ Th cell fate remains incompletely understood.

Phosphoinositide 3-kinase (PI3K) is a major cellular signaling hub that helps orchestrate T cell responses downstream of the TCR, cytokine receptors, and chemokine receptors (Okkenhaug, 2013). Coordination and amplification of T cell signaling, metabolism, and transcription are key features of PI3K. The importance of PI3K signaling in T cell differentiation is underscored by inborn errors of immunity (IEIs) in which altered PI3K signaling drives immune cell dysfunction (Lucas et al., 2016; Nunes-Santos et al., 2019; Tangye et al., 2019); activating mutations affecting the catalytic subunit of PI3Kδ cause activated PI3K delta syndrome type I (APDS1), an IEI characterized by recurrent respiratory infections, chronic EBV infection, lymphopenia, splenomegaly, and lymphadenopathy, including increased type I responses (Angulo et al., 2013; Lucas et al., 2014). Recently, it has emerged that APDS patients show a range of other clinical presentations, including Th2-driven pathologies, such as eosinophilic esophagitis, atopic dermatitis, and asthma (Bier et al., 2019; Oh et al., 2021; Tangye et al., 2019). However, both increased and reduced Th2 responses have been observed in APDS (Jia et al., 2021) and the molecular basis of T cell dysfunction, particularly in CD4^+^ Th cells, remains incompletely characterized.

Early activation of naïve CD4^+^ T cells results in the production of IL-2 and activation of STAT5, which are critical for Th1 and Th2 differentiation (Liao et al., 2011; Reem and Yeh, 1984; Yamane et al., 2005; Zhu et al., 2003). In addition to STAT5, IL-2 signaling induces activation of the mechanistic target of rapamycin complex (mTORC) 1 and mitogen-activated protein kinases (Brennan et al., 1997; Graves et al., 1992; Ray et al., 2015; Ross and Cantrell, 2018), which further promote T cell activation. Together, TCR and cytokine signals drive the induction of lineage-defining transcription factors (TFs) that specify Th cell fate, including Tbet in Th1 cells and GATA3 in Th2 cells, which in turn negatively cross-regulate one another (Zhu et al., 2006; Zhu et al., 2010). In addition, numerous negative regulators must be suppressed to permit T cell activation and differentiation. One such TF that is instrumental in maintaining naïve T cell programs is Foxo1 (Hedrick et al., 2012; Kerdiles et al., 2009; Ouyang et al., 2009). Following T cell stimulation, Foxo1 is phosphorylated in a PI3K-dependent fashion, leading to its exclusion from the nucleus and targeting for degradation (Brunet et al., 1999; Kops et al., 1999). Foxo1 inhibition switches off the naïve T cell program, allowing activated cells to take on effector characteristics. In concert with these transcriptional changes, extensive epigenetic remodeling occurs during T cell activation, which reorganizes chromatin accessibility and topology to support and maintain T cell lineage adoption (Schmidl et al., 2018; Zhao et al., 2022).

Although PI3K signaling has been extensively studied, the interplay between PI3K signaling and the transcriptional and epigenetic changes that occur during CD4 T cell differentiation remain unclear. Activation of PI3K drives the generation of phosphatidylinositol (3,4,5)-trisphosphate, leading to recruitment and activation of multiple effectors, including AKT. Activation of AKT has numerous consequences, including phosphorylation and inactivation of TFs such as Foxo1. In many cell types, PI3K and AKT are also intimately connected to the activation of mTORC1, which promotes both Th1 and Th17 differentiation, and mTORC2, which further activates AKT, and promotes both Th1 and Th2 cells (Chi, 2012; Delgoffe et al., 2009; Delgoffe et al., 2011). Accordingly, activated PI3Kδ enhances cytokine production in multiple CD4 T cell lineages (Bier et al., 2019; Preite et al., 2019). Conversely, *in vitro* polarization assays utilizing PI3K inhibitors or kinase-dead *Pik3cd*^*D910A/D910A*^ CD4 T cells show impaired Th1, Th2, and Th17 differentiation (Han et al., 2012; Okkenhaug et al., 2006; Soond et al., 2010). Whether and how PI3K differentially regulates molecular signatures in distinct CD4^+^ Th lineages remains to be explored.

Examining the consequences of activating mutations affecting *PIK3CD* offers a unique opportunity to better understand the role of PI3Kδ in CD4^+^ T cell biology. Here, we utilized a mouse model expressing the most common variant found in APDS1 (*Pik3cd*^*E1020K/+*^ mice; Preite et al., 2018) to examine Th2 responses using house dust mite (HDM) sensitization, a model of allergic airway inflammation. Unexpectedly, we found that hyperactivated PI3Kδ rewires CD4 T cell differentiation with aberrant expression of IFNγ under Th2-inducing conditions both *in vivo* and *in vitro*. This dysregulation was driven by inappropriate IL-2– and PI3Kδ-induced Foxo1 inactivation, causing a loss of Th2 lineage restriction associated with altered chromatin accessibility. Surprisingly, we linked altered CD4^+^ T cell differentiation to elevated expression of the Foxo1-repressed gene *Fasl* in *Pik3cd*^*E1020K/+*^ CD4 T cells, and Fas-mediated potentiation of TCR signaling, which occurred in the absence of the adaptor molecule FADD, a component of Fas-induced apoptosis signaling. We further provide evidence of interactions between Fas and TCR signaling components, revealed by BioID-based proximity labeling and imaging. Collectively, these data uncover nonapoptotic Fas-FasL signaling as a critical amplifier of phenotypes driven by activated PI3Kδ, thereby linking two key pathways of immune dysregulation.

## Results

### Activated PI3Kδ reshapes the HDM-induced immune response

**Figure 1.**
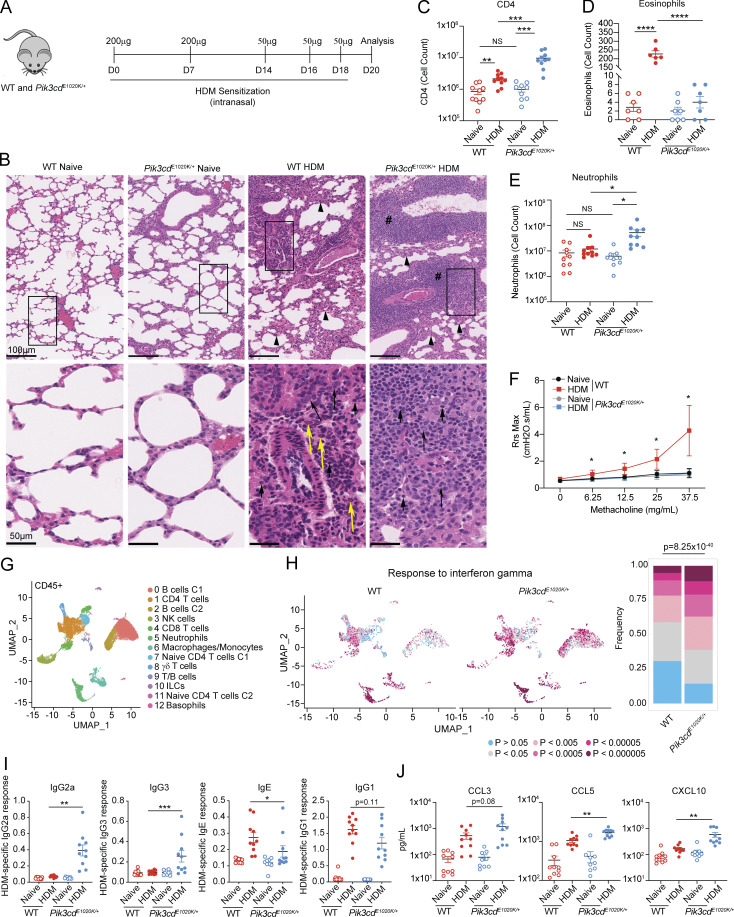
**Activated PI3Kδ reshapes the HDM-induced immune response. (A–J)** WT and *Pik3cd*^*E1020K/+*^ animals were sensitized intranasally with HDM extracts (200 μg on days 0 and 7; 50 μg on days 14, 16, and 18) and lungs examined on day 20. **(A)** Experimental outline. **(B)** H&E staining of paraffin-embedded lung sections. Top panel: Arrowheads indicate thickening of the alveolar septa; pound signs indicate iBALT. Bottom panel: Short arrows show lymphocytes, long arrows show macrophages, and yellow arrows show eosinophils. **(C)** CD4 T cell counts (liveCD45^+^TCRβ^+^CD4^+^CD8^−^) measured by flow cytometry. **(D)** Eosinophil numbers quantified from H&E-stained lung sections. **(E)** Neutrophil counts (liveLineage^−^CD11b^+^Ly6G^+^) from lungs of the indicated animals. **(F)** Airway resistance (Rrs) was measured with a flexiVent instrument as cmH2O.s/ml using the indicated concentrations of methacholine. Data in E are representative of two independent experiments with *n* = 3–4 for each group per experiment. **(G)** UMAP showing clusters of lung CD45^+^ immune cells analyzed by scRNAseq from naïve WT, naïve *Pik3cd*^*E1020K/+*^, HDM-treated WT, and HDM-treated *Pik3cd*^*E1020K/+*^ animals. Each cluster was assigned a cell type using SingleR and supervised analysis of gene expression specific to each cluster (Table S1). Cells from three mice were analyzed per genotype and condition. **(H)** Individual lung CD45^+^ immune cells identified by scRNAseq analyzed for enrichment of response to IFNγ gene set. Left: UMAP visualization of enrichment P values in lung CD45^+^ immune cells from the indicated mice; P values described in color scale below. Right: Frequencies of cells with indicated magnitudes of enrichment P values. Statistical comparison of all CD45^+^ cells from WT and *Pik3cd*^*E1020K/+*^ HDM-treated groups was performed (chi-squared test), comparing frequencies of cells with P < 0.0005. **(I)** HDM-specific IgG2a, IgG3, IgE, and IgG1 from the indicated mice. **(J)** CCL3, CCL5, and CXCL10 (pg/ml) measured from lung homogenates from the indicated mice by Luminex. Data in C–E, I, and J are from *n* = 6–10 mice for each group, pooled from two independent experiments. Data in B are representative of two independent experiments. Unless otherwise indicated, statistical comparisons were made using unpaired *t* tests. *P < 0.05, **P < 0.01, ***P < 0.001, ****P < 0.0001.

**Figure S1.**
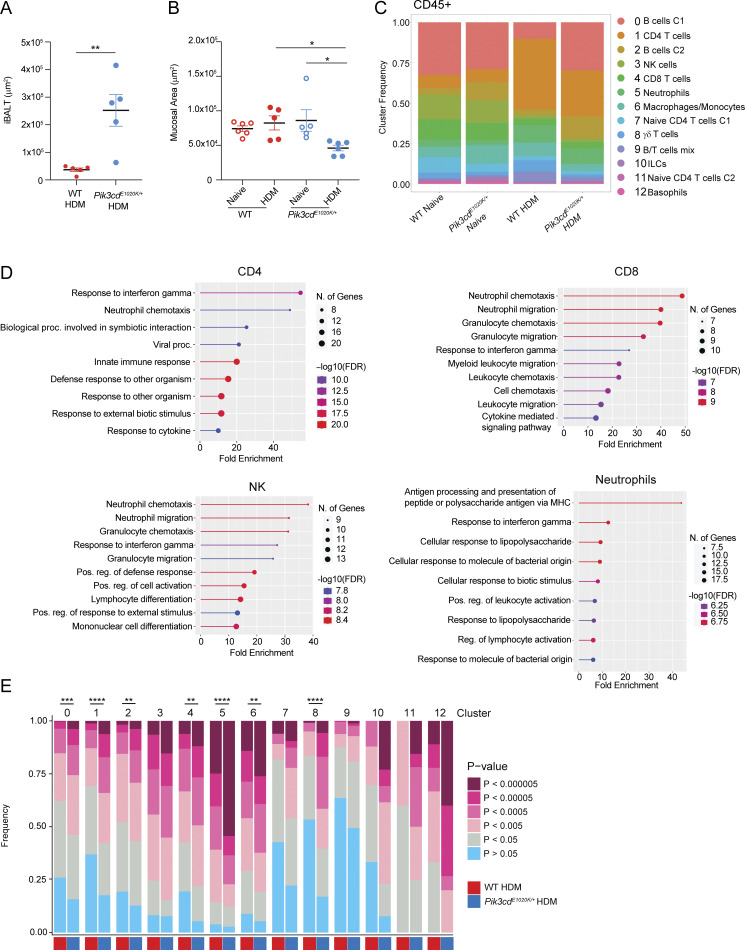
**Altered responses to HDM in *Pik3cd*** ^***E1020K/+***^ **mice.** Supporting data for Fig. 1. **(A)** iBALT area (μm^2^) measured in H&E-stained lung sections. **(B)** Mucosal area (μm^2^) measured in H&E-stained lung sections. **(A and B)***n* = 5–7 for each group, pooled from two independent experiments. **(C)** Frequencies of the indicated lung CD45^+^ immune cell populations (clusters 0–12) identified by scRNAseq from naïve WT, naïve *Pik3cd*^*E1020K/+*^, HDM-treated WT, and HDM-treated *Pik3cd*^*E1020K/+*^ animals from Fig. 1 G. **(D)** Pathway enrichment analysis using genes upregulated in HDM-treated *Pik3cd*^*E1020K/+*^ cells relative to HDM-treated WT counterparts for indicated cell types using scRNAseq data of lung CD45^+^ immune cells. Pathway enrichment was performed using ShinyGo (Ge et al., 2020). **(E)** Cluster-specific analysis of frequencies of cells with the indicated enrichment P value for the response to IFNγ gene set. Statistical comparison of WT and *Pik3cd*^*E1020K/+*^ HDM-treated groups for each cluster was performed (chi-squared test), comparing frequencies of cells with P < 0.0005. Unless otherwise indicated, statistical comparisons were made using unpaired *t* tests. *P < 0.05, **P < 0.01, ***P < 0.001, ****P < 0.0001.

To evaluate how activated PI3Kδ affects Th2 differentiation *in vivo*, we examined responses to sensitization with HDM, a model of allergic airway inflammation characterized by Th2 cell and eosinophilic infiltrates and airway hyperreactivity (Fig. 1 A). Histological staining of lung sections revealed increased immune cell infiltration in lungs of HDM-treated *Pik3cd*^*E1020K/+*^ mice relative to WT counterparts, with significantly elevated inducible bronchus-associated lymphoid tissue (iBALT) (Fig. 1 B and Fig. S1 A) and increased CD4^+^ T cells, the primary mediators of HDM-induced responses (Fig. 1 C). However, while HDM-treated WT mice showed a mixed infiltrate of lymphocytes, macrophages, and eosinophils, *Pik3cd*^*E1020K/+*^ mice had reduced numbers of eosinophils (Fig. 1 D) and elevated neutrophils compared with WT (Fig. 1 E). Lungs from HDM-treated *Pik3cd*^*E1020K/+*^ mice also exhibited a reduction of total mucosal area compared with both naïve *Pik3cd*^*E1020K/+*^ and WT HDM-treated animals, suggestive of an impaired tissue repair response (Fig. S1 B). HDM-treated WT animals showed robust airway hyperresponsiveness (AHR), with elevated airway resistance in response to increasing concentrations of methacholine (Fig. 1 F). In contrast, HDM-treated *Pik3cd*^*E1020K/+*^ lungs completely lacked an AHR signature and instead exhibited a pattern of airway resistance similar to unsensitized naïve animals. Thus, HDM induced a robust but distinct response in mice expressing activated PI3Kδ.

Single-cell RNA sequencing (scRNAseq) of total CD45^+^ cells from lungs of naïve and HDM-treated mice (Fig. 1 G and Fig. S1 C) identified 13 distinct populations of immune cells, defined by unique gene expression signatures (Table S1). For all populations, we compared differential gene expression between WT and *Pik3cd*^*E1020K/+*^ HDM-treated mice using pathway enrichment analysis. Notably, we saw a prominent enrichment of the response to IFNγ gene set, as well as a neutrophil chemotaxis signature in multiple populations from *Pik3cd*^*E1020K/+*^ mice (Fig. 1 H and Fig. S1 D). Analysis of individual cells in each cluster revealed increased frequencies of cells with highly significant enrichments (P < 0.0005) of response to IFNγ signatures in *Pik3cd*^*E1020K/+*^ lungs, including CD4 T cells (cluster 1), CD8 T cells (cluster 4), neutrophils (cluster 5), macrophage/monocytes (cluster 6), γδ T cells (cluster 8), and B cells (clusters 0 and 2) (Fig. 1 H and Fig. S1 E). Accordingly, serum from *Pik3cd*^*E1020K/+*^ mice showed significantly elevated HDM-specific IgG2a and IgG3 (Fig. 1 I), which are induced through B cell class switch recombination driven by IFNγ (Snapper et al., 1988; Snapper et al., 1992), and are not observed in WT animals following HDM treatment. Conversely, *Pik3cd*^*E1020K/+*^ serum showed reduced HDM-specific IgE with a trend toward reduced IgG1 compared with WT (Fig. 1 I). Lung homogenates from HDM-treated *Pik3cd*^*E1020K/+*^ animals also exhibited elevated concentrations of the IFNγ-induced chemokines CCL3, CCL5, and CXCL10 (Fig. 1 J). Therefore, *Pik3cd*^*E1020K/+*^ mice showed a global change in polarized responses to HDM, reshaping immunity toward a type I inflammatory response.

### Inappropriate Th1 responses in *Pik3cd*^*E1020K/+*^ lungs following HDM sensitization, at the expense of Th2 immunity

**Figure 2.**
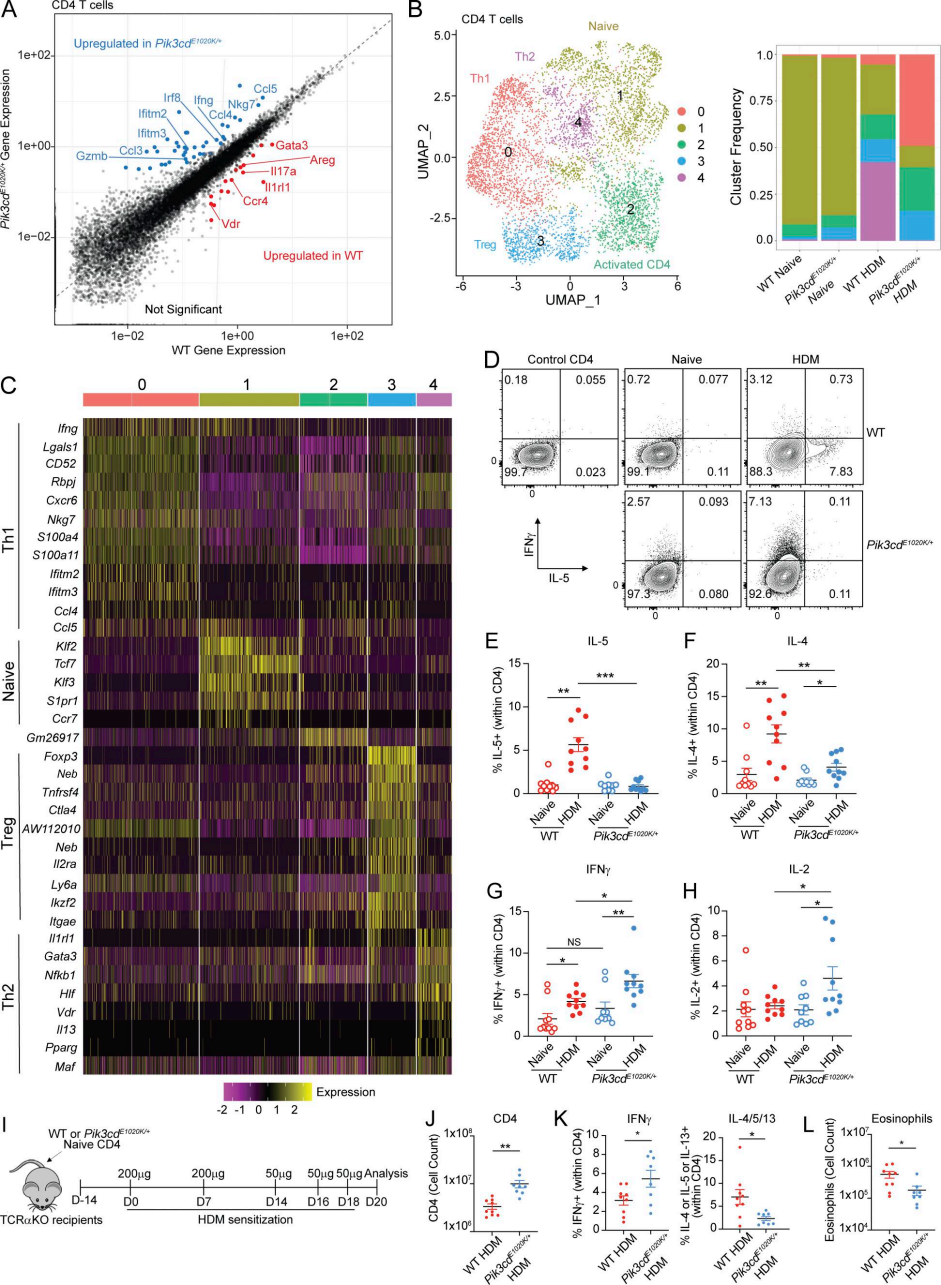
**Aberrant Th1 responses at the expense of Th2 immunity in *Pik3cd*** ^***E1020K/+***^ **lungs following HDM sensitization. (A)** Scatter plot comparing gene expression in CD4 T cells from WT and *Pik3cd*^*E1020K/+*^ HDM-treated mice (cluster 1 from Fig. 1 G). DEGs upregulated in WT CD4 T cells in red, and genes upregulated in *Pik3cd*^*E1020K/+*^ CD4 T cells in blue. **(B)** CD4^+^ T cells from scRNAseq of total CD45^+^ lung immune cells (cluster 1 from Fig. 1 G) were re-clustered to identify five unique clusters (0–4) of CD4^+^ T cells. Left: UMAP showing distribution of clusters 0–4; identities of each cluster were assigned based on cluster-specific gene expression. Right: Proportion of cells from each cluster in indicated mice. **(C)** Seurat heatmap showing expression of cluster-defining genes for indicated populations. **(A–C)** Cells from three mice were analyzed per genotype and condition. **(D)** Representative flow cytometry plots of intracellular IFNγ and IL-5 expression in lung CD4^+^ T cells (liveCD45^+^TCRβ^+^CD4^+^CD8^−^) from indicated mice. **(E–H)** Frequencies of lung CD4^+^ T cells expressing (E) IL-5; (F) IL-4; (G) IFNγ; and (H) IL-2 from indicated groups. **(D–H)***n* = 9–10 for each group, pooled from two independent experiments. **(I–L)** TCRα-deficient recipient mice were injected with 1 × 10^6^ WT or *Pik3cd*^*E1020K/+*^ naïve CD4 T cells 14 days prior to HDM sensitization. HDM was administered intranasally as indicated. *n* = 9–10 for each group, pooled from two independent experiments. **(I)** Experimental design. **(J)** Cell counts of lung CD4 T cells (liveCD45^+^TCRβ^+^CD4^+^CD8^−^) from the indicated groups. **(K)** Frequencies of IFNγ^+^ (left) and IL-4/5/13^+^ (right) lung CD4 T cells from the indicated groups. Frequencies of IL-4/5/13^+^ cells were calculated using Boolean (or) gating. **(L)** Cell counts of eosinophils (liveCD45^+^CD3^−^NK1.1^−^CD19^−^CD11b^+^Ly6G^−^SiglecF^+^) from lungs of the indicated mice. Statistical comparisons were made using unpaired *t* tests. *P < 0.05, **P < 0.01, ***P < 0.001.

**Figure S2.**
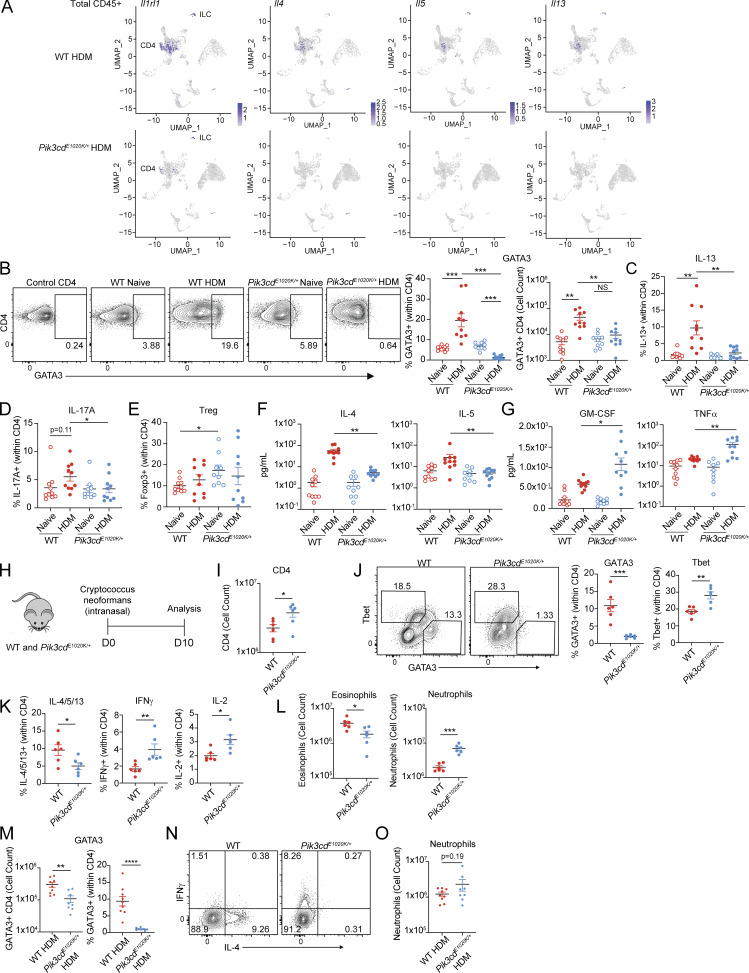
**Altered** **CD4** ^**+**^ **T cell differentiation** **in *Pik3cd*** ^***E1020K/+***^ **mice *in vivo*.** Supporting data for Fig. 2. **(A)** Feature plots showing expression of Th2-defining genes *Il1rl1*, *Il4*, *IL5*, and *Il13* in scRNAseq analyses of lung CD45^+^ immune cells from the indicated mice. Cells from three mice were analyzed by scRNAseq per genotype and condition. **(B)** Flow cytometry of lung CD4 T cells (liveCD45^+^TCRβ^+^CD4^+^CD8^−^) measuring GATA3 expression in the indicated populations. *n* = 9–10 for each group, pooled from two independent experiments. Left: Representative plots showing GATA3 and CD4 expression. Right: Frequencies (left) and cell counts (right) of GATA3^+^ CD4 T cells in the indicated groups. **(C–E)** Frequencies of (C) IL-13^+^ (liveCD45^+^TCRβ^+^CD4^+^CD8^−^ CD4^+^); (D) IL-17A^+^ (liveCD45^+^TCRβ^+^CD4^+^CD8^−^ CD4^+^), and (E) Foxp3^+^ (Treg) lung CD4 T cells (liveCD45^+^TCRβ^+^CD4^+^CD8^−^ CD4^+^) in the indicated groups, measured by intracellular staining and flow cytometry. *n* = 9–10 for each group, pooled from two independent experiments. **(F and G)** Cytokine concentrations (pg/ml) measured from lung homogenates from the indicated mice by Luminex. Th2 cytokines IL-4 and IL-5 are shown in F, and Th1 cytokines GM-CSF and TNFα are shown in G. *n* = 9–10 for each group, pooled from two independent experiments. **(H–L)** WT and *Pik3cd*^*E1020K/+*^ animals were infected intranasally with *C. neoformans*, and were sacrificed 10 days after infection, and lung tissue was harvested for analysis. *n* = 6 for each group, pooled from two independent experiments. **(H)** Experimental design. **(I)** Cell counts of CD4 T cells (liveCD45^+^TCRβ^+^CD4^+^CD8^−^ CD4^+^) from lungs of the indicated mice. **(J)** Flow cytometry of lung CD4 T cells (liveCD45^+^TCRβ^+^CD4^+^CD8^−^) measuring GATA3 and Tbet expression in the indicated populations. Left: Representative flow cytometry plots. Right: Frequencies of Tbet^+^ and GATA3^+^ CD4 T cells from the indicated groups. **(K)** Frequencies of IL-4/5/13^+^ (left), IFNγ^+^ (middle), and IL-2^+^ (right) lung CD4 T cells (liveCD45^+^TCRβ^+^CD4^+^CD8^−^ CD4^+^) in the indicated groups, measured by intracellular staining and flow cytometry. Frequencies of IL-4/5/13^+^ cells were calculated using Boolean (or) gating. **(L)** Cell counts of eosinophils (liveCD45^+^CD3^−^NK1.1^−^CD19^−^CD11b^+^Ly6G^−^SiglecF^+^) and neutrophils (liveCD45^+^CD3^−^NK1.1^−^CD19^−^CD11b^+^Ly6G^+^) from lungs of the indicated mice. **(M–O)** Supplemental data for CD4^+^ T cell transfer experiments in Fig. 2, I–L. *n* = 9–10 mice for each group, pooled from two independent experiments. **(M)** Cell counts (left) and frequencies (right) of GATA3^+^ CD4 T cells from the indicated groups. **(N)** Representative flow cytometry plots showing IFNγ and IL-4 expression in lung CD4 T cells (liveCD45^+^TCRβ^+^CD4^+^CD8^−^) from the indicated mice. **(O)** Cell counts of neutrophils (liveCD45^+^CD3^−^NK1.1^−^CD19^−^CD11b^+^Ly6G^+^) from lungs of the indicated mice. Statistical comparisons were made using unpaired *t* tests. *P < 0.05, **P < 0.01, ***P < 0.001, ****P < 0.0001.

To further understand these phenotypes, we focused on CD4 T cells, which are the primary mediators of responses to HDM. Differential gene expression analysis comparing HDM-treated WT and *Pik3cd*^*E1020K/+*^ CD4 T cells in pseudobulk (cluster 1, Fig. 1 G) revealed reduced expression of genes characteristic of pathogenic Th2 cells in *Pik3cd*^*E1020K/+*^ CD4 cells, including *Gata3*, *Il1rl1,* encoding the IL-33 receptor ST2, and *Areg*, encoding amphiregulin, a protein associated with tissue repair in asthma (Fig. 2 A); Uniform Manifold Approximation and Projections (UMAPs) of total CD45^+^ cells confirmed a severe reduction of expression of these and other key Th2 lineage–defining genes (Fig. S2 A), as well as diminished expression of IL-5 and IL-13 by ILCs. We also observed decreased IL-17A expression, despite increased neutrophil numbers (Fig. 2 A). In contrast, we observed increased expression of genes associated with a cytolytic Th1 signature in *Pik3cd*^*E1020K/+*^ CD4 cells compared with WT, including *Ifng, Gzmb*, and *Nkg7* (Fig. 2 A).

Reclustering of CD4 T cells (cluster 1, Fig. 1 G) identified two distinct subpopulations, defined by unique gene expression patterns (Fig. 2, B and C). Cluster 1 expressed signature naïve CD4 T cell genes such as *Ccr7*, *S1p1r*, and *Klf2*, and was prominent in both naïve WT and *Pik3cd*^*E1020K/+*^ animals but was reduced after HDM treatment (Fig. 2, B and C). Cluster 4, defined by expression of *Gata3*, *Il1rl1*, and *Il13* as a Th2 population (Fig. 2 C), was increased in WT CD4 cells after HDM treatment. However, this cluster was virtually absent among *Pik3cd*^*E1020K/+*^ CD4 T cells (Fig. 2 B). In contrast, cluster 0, which was defined by expression of *Ifng* and numerous Th1 signature genes, dominated among *Pik3cd*^*E1020K*^ CD4 T cells after HDM treatment, despite making up only a minor fraction of WT CD4 cells.

Flow cytometry confirmed that HDM induced a population of CD4 cells expressing GATA3 in WT lungs; however, these were significantly reduced, both in number and in frequency, in lungs of *Pik3cd*^*E1020K/+*^ mice (Fig. S2 B), as was the frequency of CD4^+^ T cells producing Th2 cytokines, IL-4, IL-5, and IL-13 (Fig. 2, D–F and Fig. S2 C). *Pik3cd*^*E1020K/+*^ mice also showed diminished IL-17A^+^ Th17 cells relative to WT counterparts (Fig. S2 D), but no differences in Foxp3^+^ Treg cells following HDM (Fig. 2 B and Fig. S2 E). In contrast, lungs from HDM-treated *Pik3cd*^*E1020K/+*^ mice showed elevated frequencies of IFNγ- and IL-2-expressing CD4^+^ T cells relative to WT (Fig. 2, G and H). Cytokine measurements from total lung homogenates confirmed significantly impaired induction of type 2 cytokines IL-4 and IL-5 (Fig. S2 F) but elevated TNFα and GM-CSF (Fig. S2 G) in *Pik3cd*^*E1020K/+*^ mice. Similar results were seen in response to *Cryptococcus neoformans* (Fig. S2 H), a fungal pathogen that induces early Th2 responses (Dang et al., 2022), where we observed increased numbers of CD4^+^ T cells (Fig. S2 I), yet significantly diminished frequencies of GATA3^+^ CD4 T cells (Fig. S2 J), type 2 cytokine-producing cells (Fig. S2 K), and eosinophils (Fig. S2 L) in lungs of *Pik3cd*^*E1020K/+*^ mice relative to WT following infection. Again, significantly elevated frequencies of Tbet^+^ cells and IFNγ-producing CD4 T cells (Fig. S2, J and K), accompanied by increased numbers of neutrophils, were observed in lungs of *Pik3cd*^*E1020K/+*^ mice (Fig. S2 L). Thus, *Pik3cd*^*E1020K/+*^ CD4^+^ T cells showed disproportionate Th1 differentiation at the expense of Th2 lineage adoption.

To evaluate whether these phenotypes were CD4 T cell intrinsic, naïve CD4 T cells from WT or *Pik3cd*^*E1020K/+*^ mice were adoptively transferred into TCRα-deficient recipients, which were then sensitized with HDM (Fig. 2 I). Like the intact animals, mice receiving *Pik3cd*^*E1020K/+*^ CD4 T cells showed increased numbers of lung CD4 T cells (Fig. 2 J), yet impaired induction of GATA3 (Fig. S2 M), diminished frequencies of CD4 cells producing type 2 cytokines (IL-4, IL-5, IL-13), and elevated frequencies of IFNγ-producing CD4 cells following HDM treatment (Fig. 2 K and Fig. S2 N). Animals receiving *Pik3cd*^*E1020K/+*^ CD4 T cells also showed reduced numbers of eosinophils (Fig. 2 L), with a trend toward increased numbers of neutrophils (Fig. S2 O), mirroring the phenotype of intact *Pik3cd*^*E1020K/+*^ mice. Defective type 2 responses in *Pik3cd*^*E1020K/+*^ mice were therefore CD4 T cell–intrinsic and not limited to HDM exposure.

### Loss of Th2 lineage restriction in *Pik3cd*^*E1020K/+*^ CD4 T cells *in vitro*

**Figure 3.**
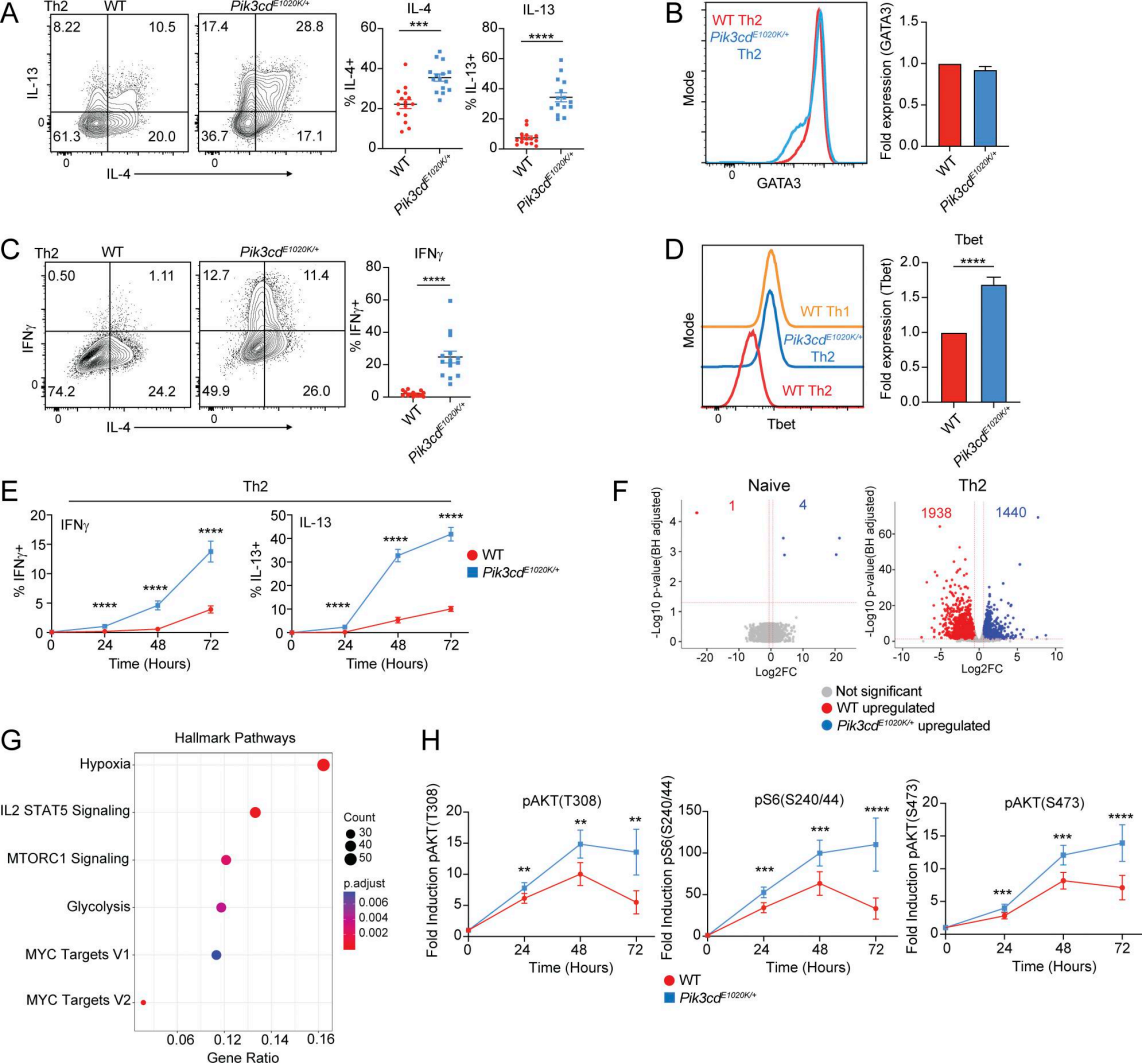
**Hyperactivated PI3Kδ disrupts Th2 lineage restriction. (A–E)** Naïve CD4 T cells were activated with αCD3 + αCD28 in the presence of WT T-depleted APCs under Th2-polarizing conditions (IL-4 + αIL-12) for 72 h. **(A)** Left: Representative flow cytometry plots showing IL-4 and IL-13 staining in Th2-polarized live CD4^+^ cells. Right: Percentages of IL-4^+^ and IL-13^+^ cells from the indicated mice. *n* = 15 for each group, from 15 independent experiments. **(B)** Left: Representative flow cytometry histograms showing GATA3 expression in Th2-polarized live CD4^+^ cells from the indicated mice. Right: Fold GATA3 expression (MFI normalized to WT) in Th2-polarized live CD4^+^ cells from the indicated mice. *n* = 14 for each group, from 14 independent experiments. **(C)** Left: Representative flow cytometry plots showing IFNγ and IL-4 staining in Th2-polarized live CD4^+^ cells. Right: Percentages of IFNγ^+^ cells. **(D)** Left: Representative flow cytometry histograms showing Tbet expression in Th2 and WT control Th1-polarized live CD4^+^ cells. Right: Fold Tbet expression (MFI normalized to WT) in Th2-polarized live CD4^+^ cells from the indicated mice. **(C and D)***n* = 14 for each group, from 14 independent experiments. **(E)** Percentages of IFNγ^+^ (left) and IL-13^+^ (right) cells over a time course of Th2 differentiation (0, 24, 48, and 72 h). *n* = 10 for each group, from 10 independent experiments. **(F)** Bulk RNAseq of WT and *Pik3cd*^*E1020K/+*^ naïve CD4 T cells and *in vitro* polarized Th2 cells. *n* = 3 biological replicates for each group. Volcano plots showing DEGs in red (WT upregulated) and blue (*Pik3cd*^*E1020K/+*^ upregulated); DEGs defined using fold change >1.5, P < 0.05. **(G)** Enrichment of hallmark pathways among DEGs comparing WT and *Pik3cd*^*E1020K/+*^ Th2 cells. **(H)** Time course of fold induction of pAKT(T308), pS6(S240/44), and pAKT(S473) in WT and *Pik3cd*^*E1020K/+*^ live CD4^+^ cells during Th2 differentiation (0, 24, 48, and 72 h), measured by flow cytometry. Fold induction calculated using MFIs of the indicated readouts normalized to the 0 time point of the corresponding genotype. *n* = 5–9 for each group, from five to nine independent experiments. Statistical comparisons were made using ratio paired *t* tests. **P < 0.01, ***P < 0.001, ****P < 0.0001.

**Figure S3.**
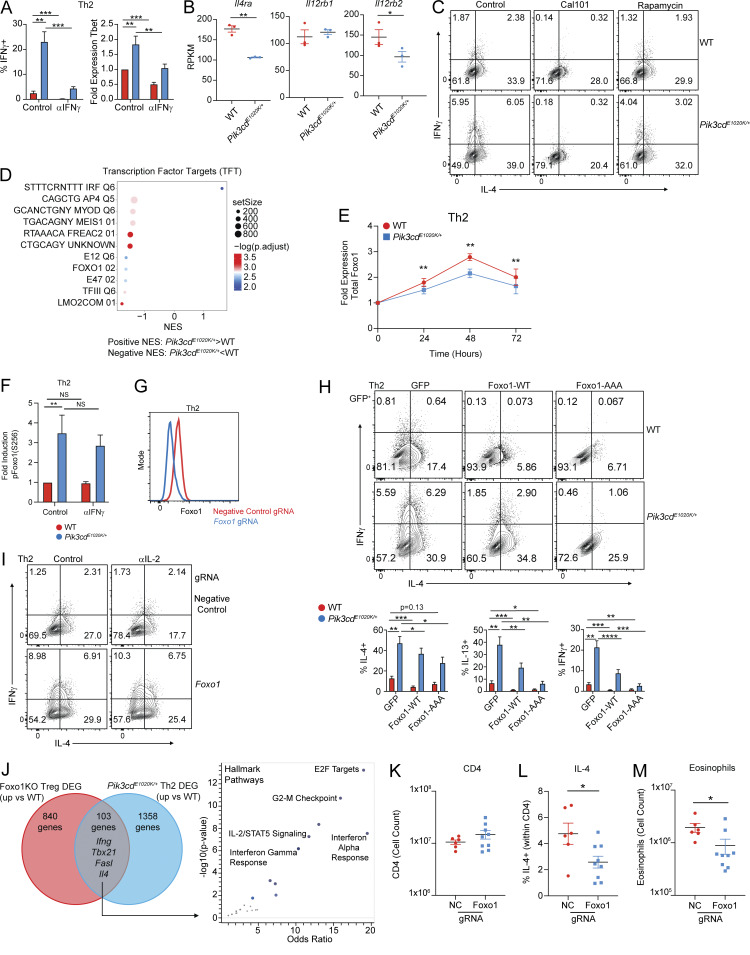
**Analyses of *in vitro* polarized Th2 cells.** Supporting data for Figs. 3, 4, and 5. **(A)** WT and *Pik3cd*^*E1020K*/+^ naïve CD4 T cells were Th2-polarized in the presence or absence of an αIFNγ blocking antibody. Left: Percentages of IFNγ^+^ cells from the indicated groups. Right: Fold Tbet expression (MFI normalized to control WT) in live CD4^+^ cells from the indicated groups. *n* = 6–8 for each group, from six to eight independent experiments. **(B)** Gene expression (RPKM) of *Il4ra*, *Il12rb1*, and *Il12rb2* in WT and *Pik3cd*^*E1020K*/+^ Th2-polarized cells. *n* = 3, bulk RNAseq. **(C)** Naïve CD4 T cells from the indicated mice were Th2-polarized for 24 h in the absence of inhibitors. At 24 h of culture, PI3Kδ inhibitor Cal101 (10 nM) or mTOR inhibitor rapamycin (200 nM) was added to polarizing media and cells were cultured for an additional 48 h. Representative flow cytometry plots showing IFNγ and IL-4 staining in live CD4^+^ cells. Data are representative of three independent experiments, *n* = 3. **(D)** GSEA comparing WT and *Pik3cd*^*E1020K/+*^ transcriptomes for expression of TFT gene sets. **(E)** Fold induction of Foxo1 expression in Th2-polarized live CD4^+^ cells, measured by flow cytometry and calculated by normalizing Foxo1 MFIs to the 0 time point of the corresponding genotype. *n* = 5 for each group, from five independent experiments. **(F)** Naïve CD4 T cells from the indicated mice were Th2-polarized in the presence or absence of αIFNγ blocking antibody, and pFoxo1(S256) was measured in live CD4^+^ cells from the indicated groups by flow cytometry. Fold induction was calculated by normalizing pFoxo1(S256) MFIs to WT control cells. *n* = 5 for each group, from five independent experiments. **(G)** Naïve CD4 T cells were nucleofected with Cas9-gRNA complexes containing NC or *Foxo1*-targeting gRNAs and underwent Th2 polarization. Representative flow cytometry histogram showing Foxo1 expression from the indicated cells, one example from nine independent experiments. **(H)** Naïve CD4 T cells from WT and *Pik3cd*^*E1020K/+*^ mice were transduced with GFP only–, Foxo1-WT– or Foxo1-AAA–encoding retroviruses under Th2-polarizing conditions. Top: Representative flow cytometry plots showing IFNγ and IL-4 expression in live GFP^+^ CD4^+^ T cells cultured under Th2-polarizing conditions from the indicated groups. Bottom, percentages of IL-4^+^ (left), IL-13^+^ (middle), and IFNγ^+^ (right) Th2-polarized liveCD4 GFP^+^ T cells from the indicated groups. *n* = 6 for each group, from six independent experiments. **(I)** WT Naïve CD4 T cells were nucleofected with NC or *Foxo1*-targeting gRNA-Cas9 complexes and polarized under Th2 conditions in the presence or absence of an IL-2 blocking antibody. Representative flow cytometry plots showing IFNγ and IL-4 expression in live CD4^+^ cells. Data are representative of two independent experiments, *n* = 5. **(J)** Comparison of transcriptomes from Foxo1 KO Treg cells and *Pik3cd*^*E1020K/+*^ Th2 cells. Genes upregulated (FC >1.5, P < 0.05) in Foxo1 KO Treg^36^ (versus WT Treg) and *Pik3cd*^*E1020K/+*^ Th2 (versus WT Th2) were compared (left), and pathway analysis (Enrichr; Xie et al., 2021) was performed on common DEGs (right). **(K–M)** Supplemental data related to Fig. 5, I and J, transfer of *Foxo1* gRNA-treated cells. **(K)** Cell counts of lung CD4 T cells (liveCD45^+^TCRβ^+^CD4^+^CD8^−^) from the indicated groups. **(L)** Frequencies of IL-4^+^ lung CD4 T cells from the indicated groups. **(M)** Cell counts of lung eosinophils (liveCD45^+^CD3^−^NK1.1^−^CD19^−^CD11b^+^Ly6G^−^SiglecF^+^) from the indicated groups. *n* = 6–9 for each group, pooled from two independent experiments. Statistical comparisons were made using ratio paired *t* tests (A, B, E, F, and H) or unpaired *t* tests (K–M). *P < 0.05, **P < 0.01, ***P < 0.001, ****P < 0.0001. NC, negative control.

To probe CD4^+^ T cell differentiation in a defined system, we examined cytokine production from isolated naïve CD4 cells that were differentiated *in vitro* in the presence of WT antigen-presenting cells (APCs). In contrast to *in vivo* responses to HDM, *Pik3cd*^*E1020K/+*^ CD4 cells differentiated *in vitro* under Th2 conditions in the presence of excess exogenous IL-4 gave rise to increased frequencies of Th2 cytokine–producing cells, including IL-4^+^ and IL-13^+^ cells (Fig. 3 A), yet with similar expression of GATA3 as WT Th2 cells (Fig. 3 B). However, *Pik3cd*^*E1020K/+*^ Th2-polarized cells also exhibited significantly elevated frequencies of IFNγ-expressing cells relative to WT Th2 cells, which showed minimal IFNγ production (Fig. 3 C). Moreover, *Pik3cd*^*E1020K/+*^ Th2 cells expressed elevated Tbet, similar to that seen in WT Th1 cells (Fig. 3 D), despite the relatively normal expression of GATA3 (Fig. 3 B). Increased IFNγ expression could be detected as early as 24 h of differentiation (Fig. 3 E) and could be prevented by IFNγ blocking antibodies (Fig. S3 A). Thus, cells expressing activated PI3Kδ showed a loss of Th2 lineage restriction *in vitro*.

### Elevated IL-2 signaling in *Pik3cd*^*E1020K/+*^ cells helps drive aberrant Th2 differentiation and heightened PI3K signaling

Bulk RNAseq analysis of *in vitro* polarized Th2 cells from WT and *Pik3cd*^*E1020K/+*^ mice revealed remarkably few differentially expressed genes (DEGs) between WT and *Pik3cd*^*E1020K/+*^ naïve CD4 T cells (Fig. 3 F). However, day 3 differentiated WT and *Pik3cd*^*E1020K/+*^ Th2 cells showed several thousand DEGs (Fig. 3 F). Analysis of *Il4ra*, *Il12rb1*, and *Il12rb2* indicated that *Pik3cd*^*E1020K/+*^ Th2 cells lacked elevated expression of Th1 and Th2 specifying cytokine receptors, suggesting that altered differentiation was not driven by abnormally high cytokine receptor expression (Fig. S3 B). However, pathway enrichment analysis of Th2-specific DEGs revealed an enrichment of the mTORC1 and MYC signaling pathways, as well as hypoxia, a pathway downstream of mTORC1 (Fig. 3 G). Time course analyses confirmed that *Pik3cd*^*E1020K/+*^ Th2 cells showed significantly elevated induction of pAKT(T308) following stimulation, as well as increased activation of both mTORC1, as evidenced by increased phosphorylation of ribosomal protein S6 (pS6(S240/44)), and mTORC2, as evidenced by increased phosphorylation of AKT on Ser473, compared with WT (Fig. 3 H). Treatment of *Pik3cd*^*E1020K/+*^ Th2-polarized cells with Cal101, a PI3Kδ-specific inhibitor, reduced cytokine production and eliminated aberrant production of IFNγ (Fig. S3 C). However, rapamycin, a mTORC1 inhibitor, only partially reduced cytokine production, with aberrant IFNγ production still present (Fig. S3 C).

**Figure 4.**
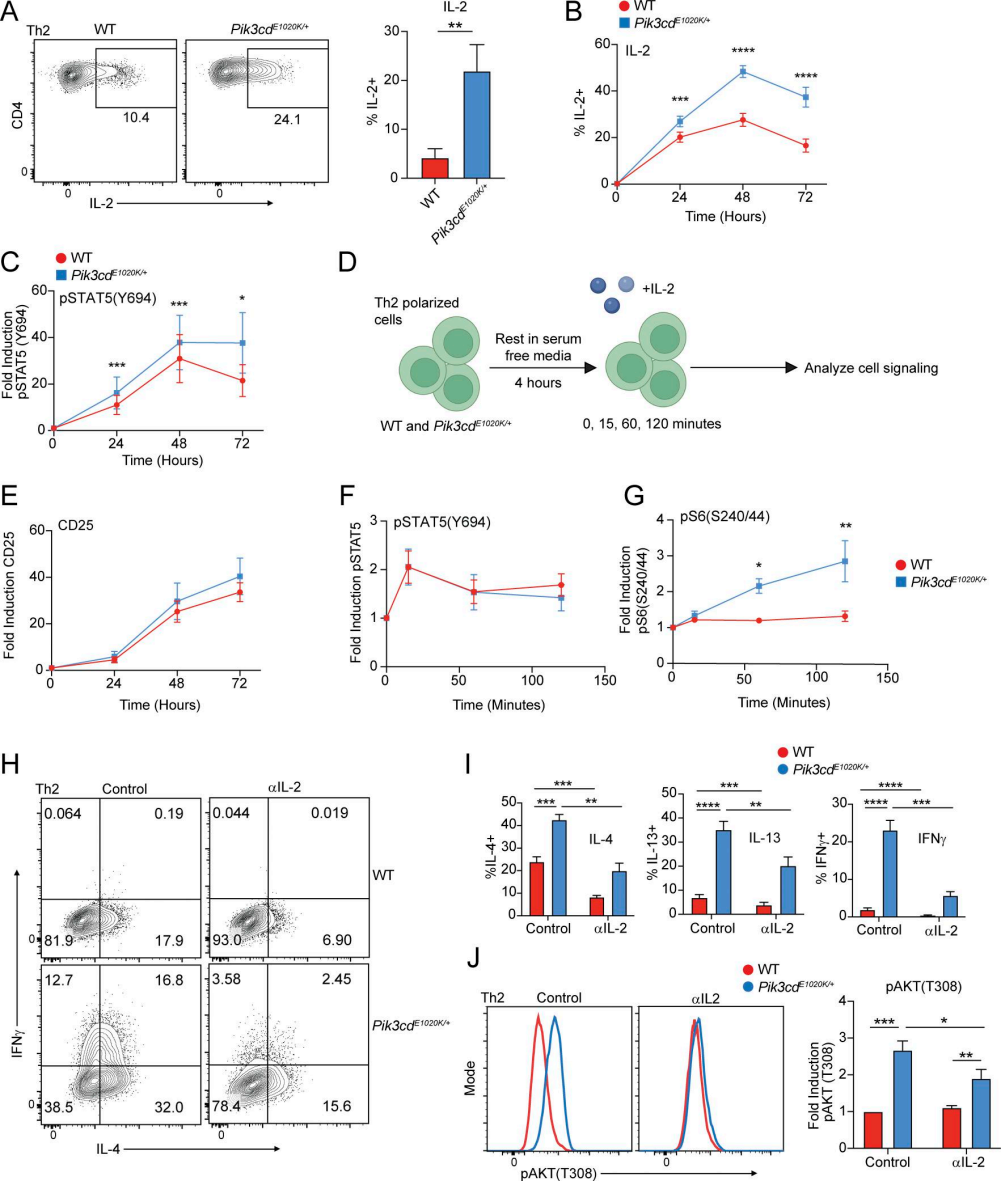
**Dysregulated IL-2 signaling rewires Th2 differentiation of *Pik3cd*** ^***E1020K*/+**^ **CD4 T cells. (A)** Left: Representative flow cytometry plots showing IL-2 and CD4 expression in Th2-polarized cells from the indicated mice. Right: Percentages of IL-2^+^ Th2 cells. *n* = 9 for each group, from nine independent experiments. **(B, C, and E)** Time course analysis (0, 24, 48, 72 h) of IL-2 production, and pSTAT5(Y694) and CD25 during Th2 polarization, measured by flow cytometry. *n* = 7–10 for each group, from 7–10 independent experiments. **(B)** Percentages of IL-2^+^ cells over time from the indicated mice. **(C)** Fold induction of pSTAT5(Y694) over time from the indicated mice. Fold induction was calculated using pSTAT5(Y694) MFIs normalized to the 0 time point of the corresponding genotype. **(D)** Schematic describing experiments shown in F and G. **(E)** Fold induction of CD25 expression from the indicated mice. Fold induction was calculated using CD25 MFIs normalized to the 0 time point of the corresponding genotype. **(F and G)** Th2-polarized CD4 T cells from WT and *Pik3cd*^*E1020K/+*^ were rested in serum-free media for 4 h and subsequently stimulated with hIL-2 over the indicated time course (0, 15, 60, 120 min). *n* = 4–5 for each group, from four to five independent experiments. **(F)** Fold induction of pSTAT5(Y694) over time from the indicated mice, measured by flow cytometry. **(G)** Fold induction of pS6(S240/44) over time from the indicated mice, measured by flow cytometry. Fold induction was calculated using MFIs (pSTAT5(Y694) or pS6(S240/44)) normalized to the 0 time point of the corresponding genotype. **(H–J)** Naïve CD4 T cells were Th2-polarized in the presence or absence of αIL-2 blocking antibody (20 μg/ml). **(H)** Representative flow cytometry plots showing IFNγ and IL-4 staining in Th2-polarized live CD4^+^ cells. **(I)** Percentages of IL-4^+^ (left), IL-13^+^ (middle), and IFNγ^+^ (right) cells from the indicated mice, in the presence or absence of αIL-2. *n* = 11 for each group, from 11 independent experiments. **(J)** Left: Representative flow cytometry plots showing pAKT(T308) in Th2-polarized live CD4^+^ cells in the presence or absence of αIL-2. Right: Fold induction of pAKT(T308) in Th2-polarized live CD4^+^ cells from the indicated groups. Fold induction was calculated by normalizing pAKT(T308) MFIs to WT control cells. *n* = 5 for each group, from five independent experiments. Statistical comparisons were made using ratio paired *t* tests. *P < 0.05, **P < 0.01, ***P < 0.001, ****P < 0.0001.

We also found an enrichment of the IL-2-STAT5 signaling pathway among Th2 DEGs (Fig. 3 G); this corresponded to increased and sustained IL-2 production and pSTAT5(Y694) in *Pik3cd*^*E1020K/+*^ Th2 cells compared with WT counterparts (Fig. 4, A–C). To determine whether *Pik3cd*^*E1020K/+*^ cells also respond more potently to IL-2 stimulation, cells were differentiated under Th2 conditions, rested in cytokine-free media, and then stimulated with IL-2 (Fig. 4 D). Over the course of Th2 differentiation, surface IL-2Ra (CD25) (Fig. 4 E) was similar between WT and *Pik3cd*^*E1020K/+*^ cells. However, although both WT and *Pik3cd*^*E1020K/+*^ cells showed similar IL-2–mediated induction of pSTAT5, *Pik3cd*^*E1020K/+*^ Th2 cells showed increased pS6(S240/44) in response to IL-2 (Fig. 4 G). Thus, *Pik3cd*^*E1020K/+*^ Th2 cells both produce more IL-2 and respond aberrantly to IL-2 stimulation.

To determine whether IL-2 contributes to altered phenotypes of *Pik3cd*^*E1020K/+*^ Th2 cells, we performed *in vitro* differentiation experiments in the presence or absence of an IL-2 blocking antibody (αIL-2) (Fig. 4, H–J). Both WT and *Pik3cd*^*E1020K/+*^ Th2-polarized cells showed significant reductions in frequencies of IL-4^+^ and IL-13^+^ cells in the presence of αIL-2, consistent with known roles of IL-2 in promoting Th2 differentiation (Ross and Cantrell, 2018) (Fig. 4, H and I). However, inappropriate IFNγ production by *Pik3cd*^*E1020K/+*^ Th2-polarized cells was also markedly reduced by IL-2 blockade, with αIL-2–treated *Pik3cd*^*E1020K/+*^ Th2 showing significantly depressed frequencies of IFNγ^+^ cells compared with control (Fig. 4, H and I). IL-2 blockade also diminished the induction of pAKT(T308) in *Pik3cd*^*E1020K/+*^ Th2 cells (Fig. 4 J), suggesting that IL-2 exacerbates PI3Kδ signaling in these cells.

### IL-2 represses Foxo1 activity in *Pik3cd*^*E1020K/+*^ CD4^+^ T cells

**Figure 5.**
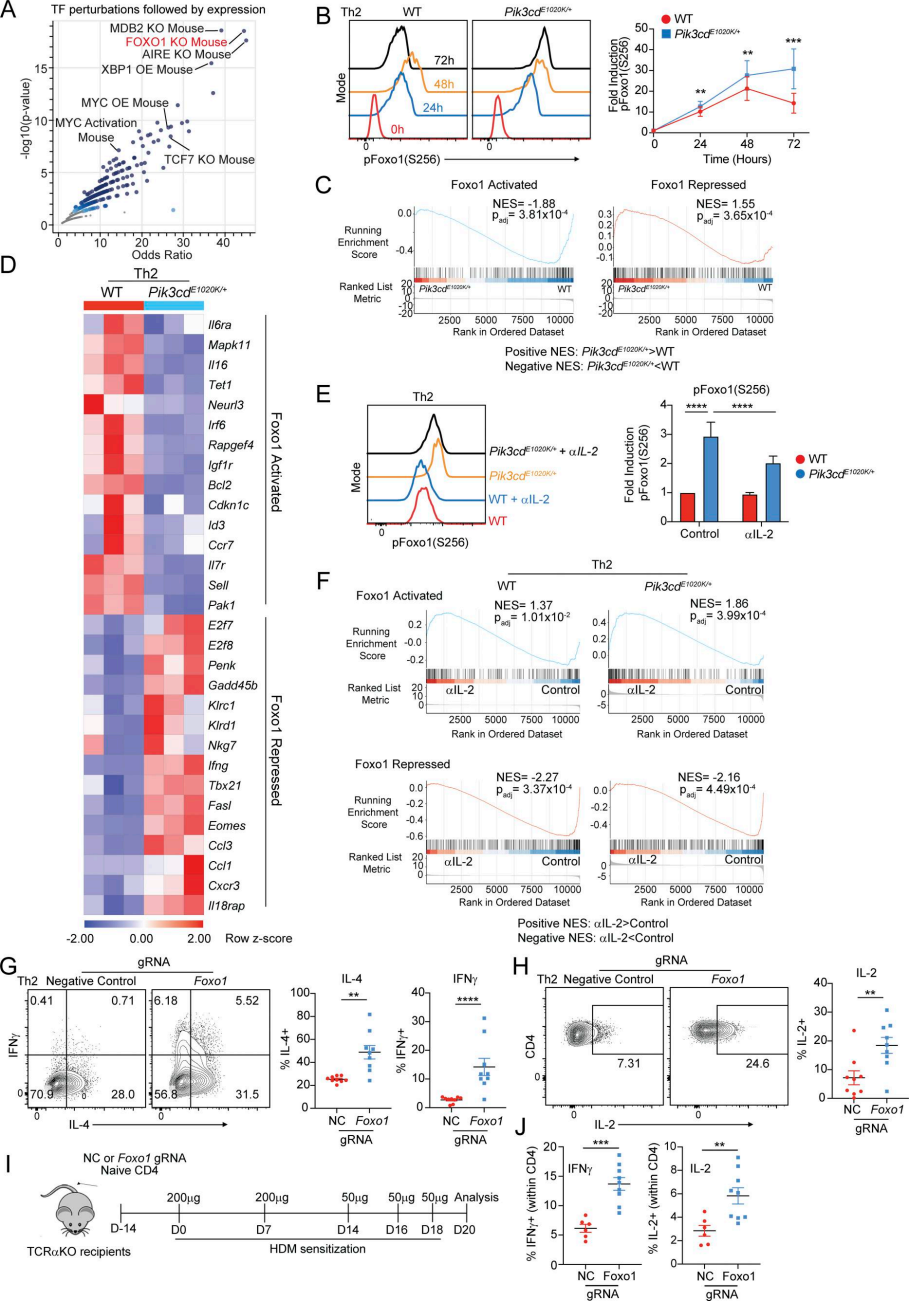
**Inactivation of Foxo1 in *Pik3cd*** ^***E1020K/+***^ **CD4** ^**+**^ **T cells impairs Th2 lineage restriction. (A)** Pathway enrichment of TF perturbations followed by expression gene sets performed using Enrichr (Xie et al., 2021): significantly enriched gene sets colored in blue. Genes upregulated in HDM-treated *Pik3cd*^*E1020K/+*^ CD4 T cells relative to WT counterparts (Fig. 2 A) were used as input for pathway enrichment. **(B)** Time course (0, 24, 48, 72 h) of pFoxo1(S256) in Th2-polarized live CD4^+^ cells from indicated mice. Left: Representative flow cytometry plots. Right: Fold induction of pFoxo1(S256) over time. Fold induction was calculated by normalizing pFoxo1(S256) MFIs to the 0 time point of the corresponding genotype. *n* = 8 for each group, from eight independent experiments. **(C)** GSEA comparing Th2-polarized WT and *Pik3cd*^*E1020K/+*^ transcriptomes for the expression of Foxo1-activated (left) and Foxo1-repressed (right) gene sets. **(D)** Gene expression heatmap (row z-score) showing normalized RPKM values of leading edge genes from Foxo1-activated and Foxo1-repressed GSEA described in Fig. 4 C. **(E)** Naïve CD4^+^ T cells from the indicated mice were Th2-polarized in the presence or absence of αIL-2 blocking antibody. Left: Representative flow cytometry plots showing pFoxo1(S256). Right: Fold induction of pFoxo1(S256). Fold induction was calculated by normalizing pFoxo1(S256) MFIs to WT control cells. *n* = 10 for each group, from 10 independent experiments. **(F)** GSEA comparing control and αIL-2–treated Th2-polarized CD4 T cell transcriptomes for the expression of Foxo1-activated (top) and Foxo1-repressed (bottom) gene sets in the indicated groups. **(G and H)** Naïve CD4 T cells were nucleofected with gRNA-Cas9 complexes containing NC or *Foxo1*-targeting gRNAs and differentiated under Th2 conditions. *n* = 9 for each group, from nine independent experiments. **(G)** Left: Representative flow cytometry plots showing IFNγ and IL-4 expression in live CD4^+^ T cells. Right: Percentages of IFNγ^+^ and IL-4^+^ cells in Th2-polarized cells. **(H)** Left: Representative flow cytometry plots showing IL-2 and CD4 expression in live CD4^+^ T cells. Right: Percentages of IL-2^+^ cells in Th2-polarized cells from the indicated groups. **(I and J)** TCRα-deficient recipient mice were injected with 1 × 10^6^ NC or *Foxo1* gRNA-Cas9–nucleofected naïve CD4 T cells 14 days prior to HDM sensitization. *n* = 6–9 for each group, pooled from two independent experiments. **(I)** Experimental design. **(J)** Frequencies of IFNγ^+^ (left) and IL-2^+^ (right) lung CD4 T cells. Statistical comparisons used ratio paired *t* tests (B and E–G) or unpaired *t* tests (I). **P < 0.01, ***P < 0.001, ****P < 0.0001. NC, negative control.

To explore potential TFs regulated by activated PI3Kδ during Th2 differentiation, we performed gene set enrichment analysis (GSEA) comparing WT and *Pik3cd*^*E1020K/+*^ cells for TF target (TFT) gene expression (Fig. S3 D). We observed significant enrichment of many more TFT gene sets in WT cells compared with *Pik3cd*^*E1020K/+*^ including AP4, MYOD, MEIS1, and genes regulated by Foxo1 (Fig. S3 D). Notably, analysis of DEGs upregulated in *Pik3cd*^*E1020K/+*^ CD4 T cells following HDM treatment *in vivo* also showed significant enrichment of a Foxo1 knockout (KO) signature (Fig. 5 A). TCR-mediated activation of PI3Kδ leads to AKT-mediated phosphorylation and inactivation of Foxo1; pFoxo1 was observed in response to TCR stimulation in both WT and *Pik3cd*^*E1020K/+*^ CD4 cells (Fig. 5 B). However, while Foxo1 phosphorylation subsided in WT Th2 cells by 72 h of stimulation, pFoxo1(S256) remained high in *Pik3cd*^*E1020K/+*^ cells (Fig. 5 B), with decreased total Foxo1 protein in *Pik3cd*^*E1020K/+*^ Th2 cells over the differentiation period (Fig. S3 E).

Using publicly available transcriptomic data, we generated lists of known Foxo1- activated and Foxo1-repressed genes (Ouyang et al., 2012) and compared transcriptomes of WT versus *Pik3cd*^*E1020K/+*^ cells by GSEA (Fig. 5 C and Table S2). While WT cells exhibited significantly enriched expression of Foxo1-activated genes, *Pik3cd*^*E1020K/+*^ cells showed significant enrichment of Foxo1-repressed gene signatures relative to WT (Fig. 5 C). Evaluation of leading-edge genes identified by GSEA (Fig. 5 D) revealed multiple relevant Foxo1-activated genes were enriched in WT cells including *Sell*, *Ccr7*, *Il7r*, and *Bcl2*. Conversely, Foxo1-repressed genes that were enriched in *Pik3cd*^*E1020K/+*^ cells had a notable type I immune response signature, including *Ifng*, *Tbx21*, *Eomes*, and *Fasl*.

To evaluate whether IL-2 signaling influenced Foxo1 activity, we measured pFoxo1(S256) following differentiation in the presence or absence of an IL-2 blocking antibody (Fig. 5 E). *Pik3cd*^*E1020K/+*^ cells showed reduced pFoxo1(S256) in αIL-2 conditions compared with control, although pFoxo1(S256) was still elevated compared with WT cells, consistent with broad effects of activated PI3Kδ. In contrast, blocking IFNγ did not significantly alter phosphorylation of Foxo1 (Fig. S3 F), although it decreased high expression of Tbet and IFNγ in *Pik3cd*^*E1020K/+*^ Th2 cells (Fig. S3 A).

We next examined whether IL-2 signaling regulates Foxo1 transcriptional activity. GSEA comparison of transcriptomes of Th2 cells differentiated in the presence or absence of αIL-2 (Fig. 5 F) showed significant enrichment of Foxo1-activated gene signatures in both αIL-2–treated WT and *Pik3cd*^*E1020K/+*^ Th2 cells, compared with controls. Conversely, both control WT and *Pik3cd*^*E1020K/+*^ Th2 cells showed significant enrichment of Foxo1-repressed genes relative to αIL-2–treated cells under Th2 conditions, suggesting IL-2 contributes to the inhibition of Foxo1 transcriptional activity in CD4 T cells.

### Foxo1 is critical for Th2 lineage restriction

To address whether altered Foxo1 regulation directly contributes to aberrant CD4 T cell differentiation, we targeted *Foxo1* using Cas9 ribonucleoprotein (RNP) complexes in naïve CD4 T cells prior to *in vitro* Th2 polarization (Fig. S3 G). *Foxo1*-targeted Th2-polarized cells showed elevated frequencies of IL-4^+^ cells, relative to negative controls (Fig. 5 G). Notably, Foxo1-deficient Th2 cells also showed aberrant production of IFNγ, recapitulating the phenotype of *Pik3cd*^*E1020K/+*^-polarized cells. Conversely, retroviral-mediated expression of Foxo1-WT or Foxo1-AAA, a Foxo1 mutant resistant to AKT-mediated phosphorylation, decreased Th2 cytokine production, especially in WT cells (Fig. S3 H), and markedly reduced the aberrant IFNγ production by *Pik3cd*^*E1020K/+*^ Th2-polarized cells. Thus, Foxo1 activity appears to be critical to prevent inappropriate IFNγ expression in Th2 cells. Foxo1-deficient Th2 cells also showed increased frequencies of IL-2^+^ cells compared with controls (Fig. 5 H). However, αIL-2 treatment of *Foxo1*-targeted Th2-polarized cells did not dampen inappropriate IFNγ production (Fig. S3 I), suggesting that the major effects of Foxo1 inhibition functioned downstream of IL-2. Together, these results highlight a signaling amplification loop whereby increased IL-2 production in activated PI3Kδ cells further promotes Foxo1 phosphorylation and inactivation, leading to a loss of Th2 lineage restriction.

Given the nearly identical Th2 phenotypes of Foxo1-deficient and *Pik3cd*^*E1020K/+*^ Th2 cells, we compared gene expression programs regulated by Foxo1 and PI3Kδ. Using publicly available transcriptomic data (Ouyang et al., 2012) from Foxo1-deficient Treg cells and our RNAseq analysis of *Pik3cd*^*E1020K/+*^ Th2 cells, we compared upregulated genes (relative to WT) from both genotypes (Fig. S3 J). From a total of 2,301 DEGs, we observed only 103 genes that were similarly upregulated in both Foxo1-deficient and *Pik3cd*^*E1020K/+*^ CD4 T cells (Fig. S3 J), consistent with broader roles of PI3Kδ and Foxo1 that are independent of one another (Spinelli et al., 2021). However, of the common DEGs, we observed multiple genes central to the phenotypes observed in altered Th2 differentiation, including *Ifng*, *Tbx21*, *Fasl*, and *IL4*. Furthermore, pathway enrichment analysis of common DEGs showed significant enrichment of IL-2/STAT5 signaling and IFNγ response signatures (Fig. S3 J), consistent with dysregulation of these pathways in both Foxo1-deficient and activated PI3Kδ Th2 cells.

To assess the importance of Foxo1 in CD4 T cell differentiation *in vivo*, we adoptively transferred negative control and *Foxo1*-targeted naïve CD4 T cells into TCRα KO recipient mice and performed HDM sensitization (Fig. 5 I). In contrast to *Pik3cd*^*E1020K/+*^ mice, animals receiving Foxo1-deficient CD4 T cells showed similar numbers of CD4 T cells as negative control counterparts, suggesting that Foxo1 inactivation does not account for the PI3Kδ-mediated increase in CD4 T cell expansion (Fig. S3 K). However, *Foxo1*-guide RNA (gRNA)–treated CD4 T cells showed significantly elevated frequencies of IFNγ^+^ cells, as well as IL-2^+^ cells (Fig. 5 J), mirroring the phenotypes of altered cytokine production by *Pik3cd*^*E1020K/+*^ CD4 T cells. We also observed reduced frequencies of IL-4^+^ CD4 T cells (Fig. S3 L), and decreased numbers of eosinophils (Fig. S3 M), in animals receiving *Foxo1*-gRNA–treated CD4 T cells compared with negative control counterparts. Thus, Foxo1 appears to be a major restriction factor for CD4 T cell cytokine production and Th2 lineage restriction both *in vivo* and *in vitro*.

### *Pik3cd* ^*E1020K*^ reshapes the epigenetic landscape of CD4^+^ T cells

**Figure 6.**
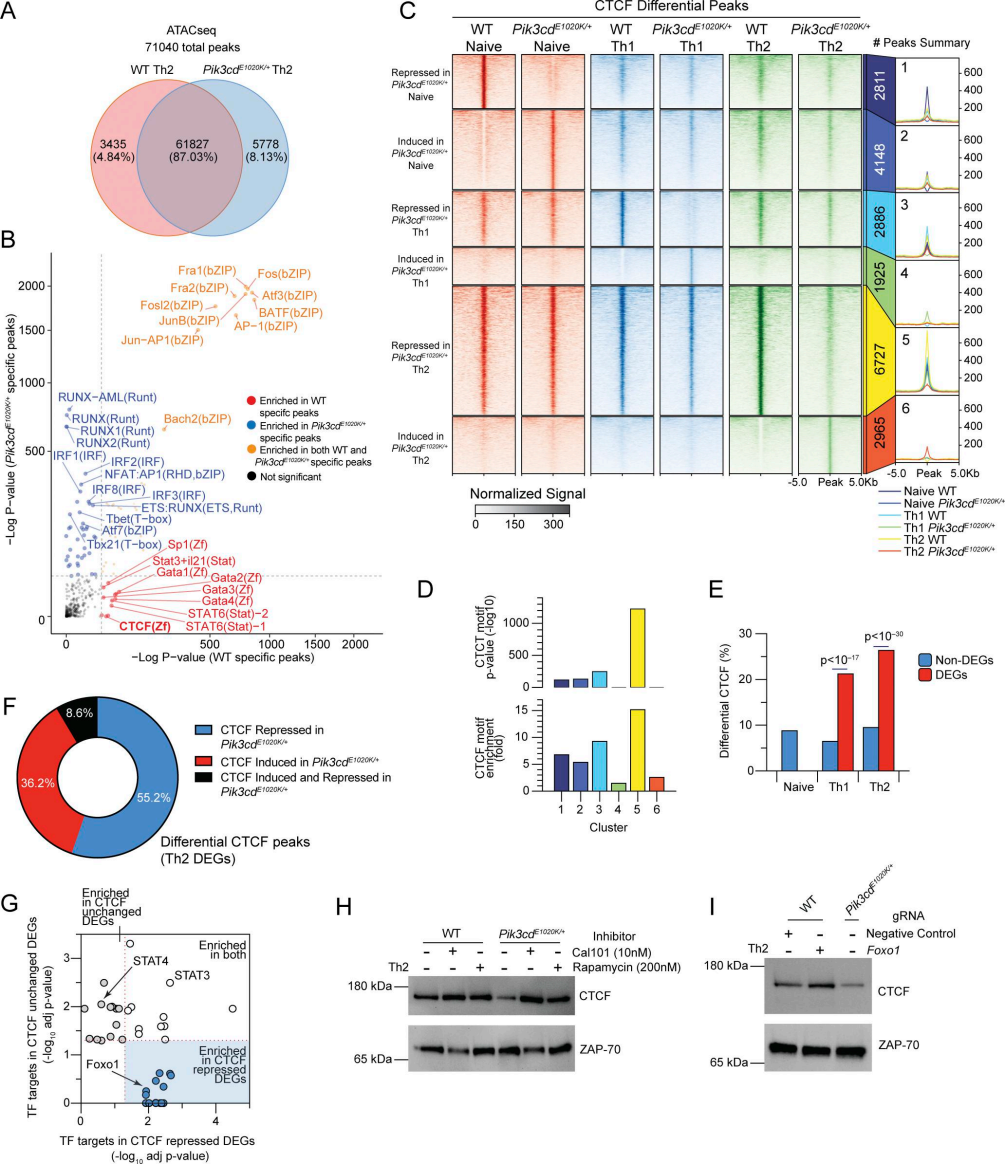
***Pik3cd*** ^***E1020K***^ **reshapes the epigenetic landscape of CD4** ^**+**^ **T cells. (A and B)** Naïve CD4 T cells from WT and *Pik3cd*^*E1020K/+*^ mice were polarized under Th2 conditions and evaluated by ATACseq (*n* = 3). A total of 71,040 peaks were detected. **(A)** Venn diagram of WT-specific, *Pik3cd*^*E1020K/+*^-specific, and common peaks. **(B)** WT and *Pik3cd*^*E1020K/+*^-specific peaks examined by motif enrichment analysis. Enrichment P values were plotted for both groups. Red: motifs specifically enriched in WT peaks; blue: motifs specifically enriched in *Pik3cd*^*E1020K/+*^ peaks; orange: motifs enriched in both groups. **(C)** Peak heatmap of CTCF CUT&Tag peaks from WT and *Pik3cd*^*E1020K/+*^ naïve, Th1, and Th2 cells organized into six clusters (1–6) specific to each indicated population, as described. **(D)** CTCF motif enrichment P value (top) and fold enrichment (bottom) in clusters 1–6. **(E)** DEGs (WT vs *Pik3cd*^*E1020K/+*^) and non-DEGs from bulk RNAseq data (Fig. 3 C) were compared in the indicated populations for percentages of genes showing differential CTCF peaks (WT versus *Pik3cd*^*E1020K/+*^). **(F)** Frequencies of CTCF peaks repressed, induced, or both induced and repressed in *Pik3cd*^*E1020K/+*^ Th2 cells (versus WT Th2) near DEGs (WT Th2 versus *Pik3cd*^*E1020K/+*^ Th2). **(G)** Th2 DEGs (WT Th2 versus *Pik3cd*^*E1020K/+*^ Th2) were organized into two categories: DEGs showing no change in CTCF (WT versus *Pik3cd*^*E1020K/+*^) and DEGs showing repressed CTCF peaks in *Pik3cd*^*E1020K/+*^ relative to WT. Pathway enrichment analysis (Enrichr; Xie et al., 2021) of TFTs (ChEA; Lachmann et al., 2010) was performed using these two categories of DEGs. Adjusted P values (−log_10_) of the top 25 enriched TF signatures in each category plotted against each other. **(H)** Western blot evaluating CTCF and Zap70 in lysates from WT and *Pik3cd*^*E1020K/+*^ Th2-polarized cells, cultured in the presence or absence of Cal101 (10 nM) or rapamycin (200 nM). Data are representative of three independent experiments (*n* = 3), quantified in Fig. S4 C. **(I)** Western blot evaluating CTCF and Zap70 in lysates from Th2-polarized NC and *Foxo1* gRNA-Cas9–nucleofected WT CD4 T cells, compared with *Pik3cd*^*E1020K/+*^ cells. Data are representative of three independent experiments (*n* = 3), Fig. S4 D. NC, negative control. Source data are available for this figure: SourceData F6.

**Figure S4.**
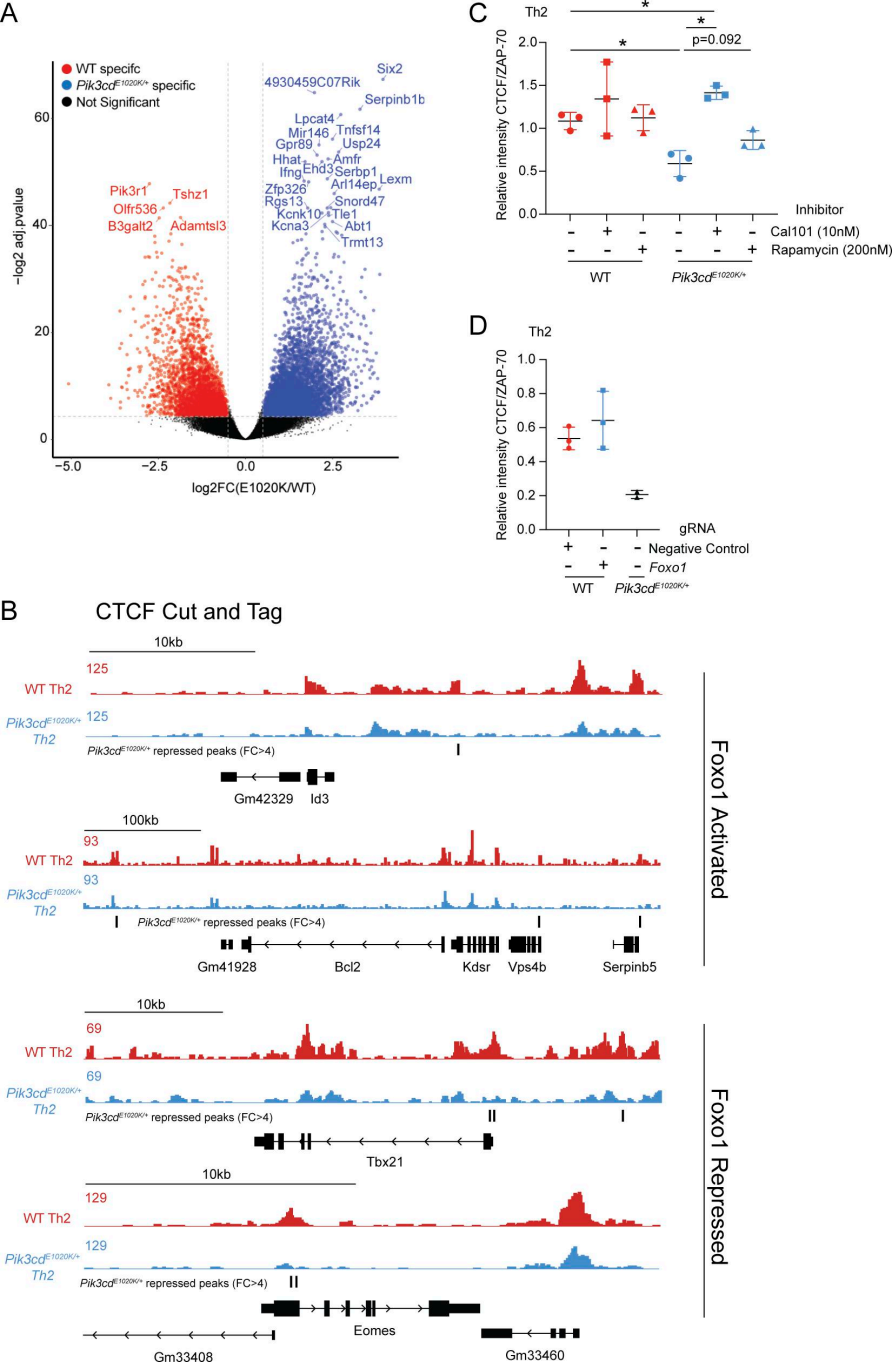
**Altered chromatin accessibility and CTCF activity in *Pik3cd*** ^***E1020K/+***^ **Th2 cells.** Supporting data for Fig. 6. **(A and B)** Naïve CD4 T cells from WT and *Pik3cd*^*E1020K/+*^ mice underwent Th2 polarization and were examined by ATACseq. *n* = 3. Volcano plot showing WT-specific peaks in red and *Pik3cd*^*E1020K/+*^-specific peaks in blue (fold change >1.5, P < 0.05) (B) CTCF CUT&Tag tracks of *Id3*, *Bcl2*, *Tbx21*, and *Eomes* loci. **(C)** Quantification of CTCF protein expressed as a ratio of CTCF/Zap70. Supporting data for Fig. 5 H. *n* = 3 for each group, from three independent experiments. **(D)** Quantification of CTCF protein expressed as a ratio of CTCF/Zap70. Supporting data for Fig. 5 I. *n* = 3 for each group, from three independent experiments. Statistical comparisons were made using ratio paired *t* tests. *P < 0.05.

To probe downstream consequences of activated PI3Kδ, we evaluated changes in chromatin by ATACseq analysis of *in vitro* differentiated WT and *Pik3cd*^*E1020K/+*^ Th2 cells. Approximately 13% of their epigenomes were differentially accessible (Fig. 6 A and Fig. S4 A). Major TF motif families that were distinct among *Pik3cd*^*E1020K/+*^ Th2-specific peaks included those binding NFAT:AP1 complexes, IFN regulatory factors (IRFs), and runt-related TFs (Runx), which are all associated with T cell activation and type I responses, as well as motifs binding T-box family members, including Tbet, reflecting their altered Th1 cytokine expression (Fig. 6 B). In contrast, WT Th2-specific peaks showed enrichment of motifs associated with Th2-promoting factors, including STAT6 and GATA3 (Fig. 6 B). However, Foxo1 motifs were not enriched in either WT- or *Pik3cd*^*E1020K/+*^-specific peaks. Of note, in addition to these lineage- and activation-defining TFs, CTCF motifs were also differentially accessible, with a strong enrichment in WT Th2-specific peaks. CTCF functions as a global regulator of chromatin organization (Zhao et al., 2022), suggesting broad alterations associated with activated PI3Kδ.

Given these observations, we employed CUT&Tag to specifically examine genome-wide CTCF binding in WT and *Pik3cd*^*E1020K/+*^ naïve CD4, Th2, and Th1 cells. Differential CTCF peaks, defined as at least fourfold difference in peak intensity, were grouped into six clusters, representing differential peaks between WT and *Pik3cd*^*E1020K/+*^ CD4 cells in naïve, and Th1- and Th2-polarized states (Fig. 6 C). We identified ∼7,000, 5,000, and 10,000 differential CTCF peaks in the naïve, Th1, and Th2 states, respectively, indicating significant genome-wide differences in CTCF binding in *Pik3cd*^*E1020K/+*^ CD4 cells; Th2 cells showed the greatest number of peaks reduced in *Pik3cd*^*E1020K/+*^ cells (Fig. 6 C). We then quantified the enrichment of CTCF motifs within the peaks in each cluster. WT Th1 (cluster 3)- and Th2 (cluster 5)-specific peaks displayed strong CTCF motif enrichment (Fig. 6 D). In contrast, *Pik3cd*^*E1020K/+*^ Th1- and Th2-specific peaks (clusters 4 and 6, respectively) exhibited surprisingly low CTCF motif enrichment (Fig. 6 D).

We next annotated differential CTCF peaks (Fig. 6 C) to genes and compared those associated with DEGs to non-DEGs (WT versus *Pik3cd*^*E1020K/+*^ Th2 cells). DEGs showed significantly higher percentages of genes associated with differential CTCF peaks relative to non-DEGs, indicating changes in CTCF profiles were enriched at loci exhibiting differential gene expression (Fig. 6 E). We next considered CTCF peaks near DEGs and determined the pattern of CTCF binding in the vicinity of these genes (Fig. 6 F); the majority of DEGs with differential CTCF peaks demonstrated a loss of CTCF binding in *Pik3cd*^*E1020K/+*^ Th2 cells (Fig. 6 F). To determine whether particular TF signatures were associated with repressed CTCF in *Pik3cd*^*E1020K/+*^cells, we compared TFTs enriched in DEGs associated with repressed CTCF peaks in *Pik3cd*^*E1020K/+*^ Th2 cells with DEGs showing no change in CTCF (Fig. 5 G). Notably, DEGs associated with repressed CTCF in *Pik3cd*^*E1020K/+*^ Th2 cells showed significant enrichment of targets for several TFs (Table S3), including Foxo1. Indeed, analysis of genomic regions surrounding both Foxo1-activated and Foxo1-repressed loci revealed altered patterns of CTCF binding in *Pik3cd*^*E1020K/+*^ Th2 cells (Fig. S4 B), suggesting broad dysregulation of chromatin.

**Figure S5.**
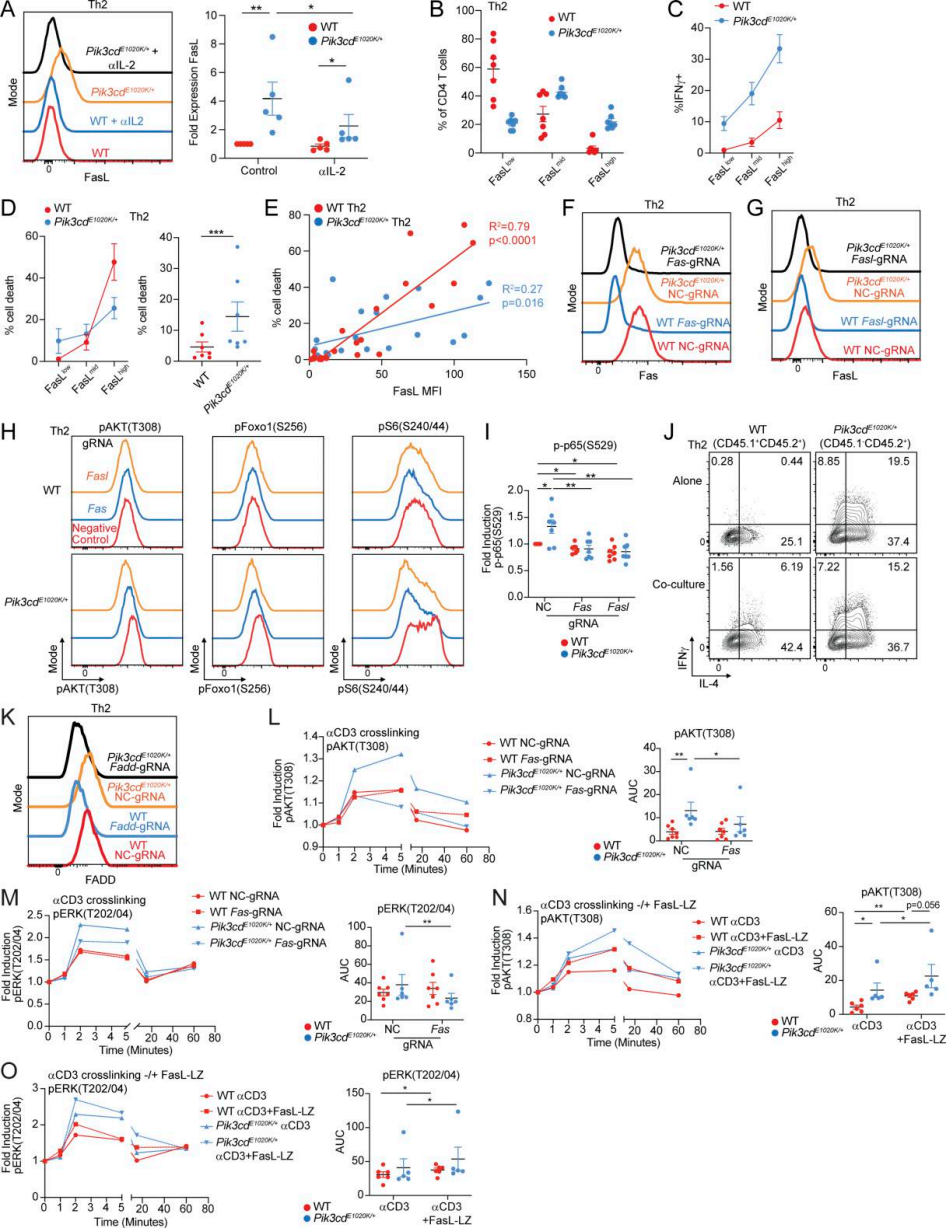
**Fas-FasL signaling potentiates T cell activation.** Supporting data for Figs. 7, 8, and 9. **(A)** Naïve CD4^+^ T cells from the indicated mice were Th2-polarized in the presence or absence of αIL-2 blocking antibody, and surface FasL expression was measured by flow cytometry. Left: Representative flow cytometry histograms of surface FasL. Right: Fold FasL expression (MFI normalized to WT control) on Th2-polarized cells from the indicated groups. *n* = 5 for each group, from five independent experiments. **(B and C)** Supplemental data for Fig. 7 C. **(B)** Frequencies of FasL^low^, FasL^mid^, and FasL^high^ live CD4 T cells from the indicated groups. *n* = 7 for each group, from seven independent experiments. **(C)** Frequencies of IFNγ^+^ cells in the indicated FasL expression category from the indicated mice. *n* = 7 for each group, from seven independent experiments. **(D)** Frequencies of dead Th2-polarized cells (gated as total CD4^+^) from the indicated groups. Left: Frequencies of dead cells within FasL^low^, FasL^mid^, and FasL^high^. Right: Frequencies of dead Th2 cells among total CD4^+^ cells. *n* = 7 for each group, from seven independent experiments. **(E)** Linear regression analyses comparing FasL MFIs with percentages of dead cells from the indicated groups. Correlation R^2^ and P values are indicated. **(F and G)** Naïve CD4 T cells from WT and *Pik3cd*^*E1020K/+*^ mice were nucleofected with Cas9-gRNA complexes containing NC, or *Fas*- or *Fasl*-targeting gRNAs and underwent Th2 polarization. **(F)** Representative flow cytometry histograms showing surface Fas expression in the indicated groups. **(G)** Representative flow cytometry histograms showing surface FasL expression in the indicated groups. **(H)** Representative flow cytometry histograms showing pAKT(T308), pFoxo1(S256), and pS6(S240/44) staining in NC, *Fas*, and *Fasl* gRNA-targeted Th2-polarized live CD4^+^ T cells from the indicated mice. Supporting data for Fig. 7 F. *n* = 8–10 for each group, from 8 to 10 independent experiments. **(I)** Fold induction of p-p65(S529) in Th2-polarized live CD4 T cells from the indicated groups, measured by flow cytometry. For all readouts, fold induction was calculated by normalizing MFIs to NC WT cells. *n* = 7 for each group, from seven independent experiments. **(J)** WT (CD45.1^+^CD45.2^+^) and *Pik3cd*^*E1020K/+*^ (CD45.1^−^CD45.2^+^) naïve CD4 T cells were Th2-polarized either alone (top) or cocultured (bottom) at a 1:1 ratio. Representative flow cytometry plots showing IFNγ and IL-4 expression in live CD4^+^ cells. Data are representative of six independent experiments. **(K)** Supporting data for Fig. 8, A–C. Representative flow cytometry histograms showing FADD staining in NC and *Fadd* gRNA-Cas9–treated cells from the indicated groups. **(L and M)** Naïve CD4^+^ T cells from WT and *Pik3cd*^*E1020K/+*^ mice were nucleofected with Cas9-gRNA complexes containing NC or *Fas*-targeting gRNAs and underwent αCD3/CD28 stimulation in the presence of hIL-2 for 72 h. Cells were subsequently rested in serum-free media, underwent αCD3 (1 μg/ml) crosslinking over a time course (0, 1, 2, 5, 15, 60 min), and were analyzed by flow cytometry. *n* = 6–7 for each group, from six to seven independent experiments. **(L)** Fold induction of pAKT(T308) and (M) pERK(T202/04) over time for the indicated groups. Fold induction was calculated using MFIs normalized to the 0 time point of the corresponding sample. Right: AUC quantification of pAKT(T308) and pERK(T202/04) time courses for the indicated groups. **(N and O)** Naïve CD4^+^ T cells from WT and *Pik3cd*^*E1020K/+*^ mice underwent αCD3/CD28 stimulation in the presence of hIL-2 for 72 h. Cells were subsequently rested in serum-free media, underwent αCD3 (1 μg/ml) crosslinking in the presence or absence of FasL-LZ over a time course (0, 1, 2, 5, 15, 60 min), and were analyzed by flow cytometry. *n* = 6–7 for each group, from six to seven independent experiments. **(N)** Fold induction of pAKT(T308) and (O) pERK(T202/04) over time for the indicated groups. Fold induction was calculated using MFIs normalized to the 0 time point of the corresponding sample. Right: AUC quantification of pAKT(T308) and pERK(T202/04) time courses for the indicated groups. Statistical comparisons were made using ratio paired *t* tests, unless otherwise indicated. *P < 0.05, **P < 0.01, ***P < 0.001. AUC, area under the curve; NC, negative control.

Given the strong loss of CTCF-DNA binding in *Pik3cd*^*E1020K/+*^ CD4 T cells, we examined CTCF protein expression (Fig. 6 H). *Pik3cd*^*E1020K/+*^ Th2 cells demonstrated reduced CTCF protein compared with WT counterparts (Fig. 6 H and Fig. S4 C). Furthermore, treatment of *Pik3cd*^*E1020K/+*^ Th2-polarized cells with Cal101, a PI3Kδ-specific inhibitor, restored CTCF protein to levels exceeding control WT cells, confirming a role of PI3Kδ in regulating CTCF expression (Fig. 6 H and Fig. S5 C). Inhibition of mTOR signaling via rapamycin also increased CTCF protein expression in *Pik3cd*^*E1020K/+*^ Th2-polarized cells compared with controls, but to a lesser extent (Fig. 6 H and Fig. S4 C). However, Foxo1-deficient Th2-polarized cells did not show reduced CTCF expression, having similar CTCF expression as WT cells (Fig. 6 I and Fig. S4 D). Thus, although activated PI3Kδ was associated with diminished CTCF protein and repressed CTCF profiles, reduced CTCF protein was not recapitulated by Foxo1 repression, suggesting that loss of CTCF was a Foxo1-independent effect of activated PI3Kδ and therefore likely not required for altered differentiation and cytokine patterns in activated PI3Kδ and Foxo1-deficient cells.

### Fas-FasL signaling drives PI3Kδ hyperactivation in Th2 cells

**Figure 7.**
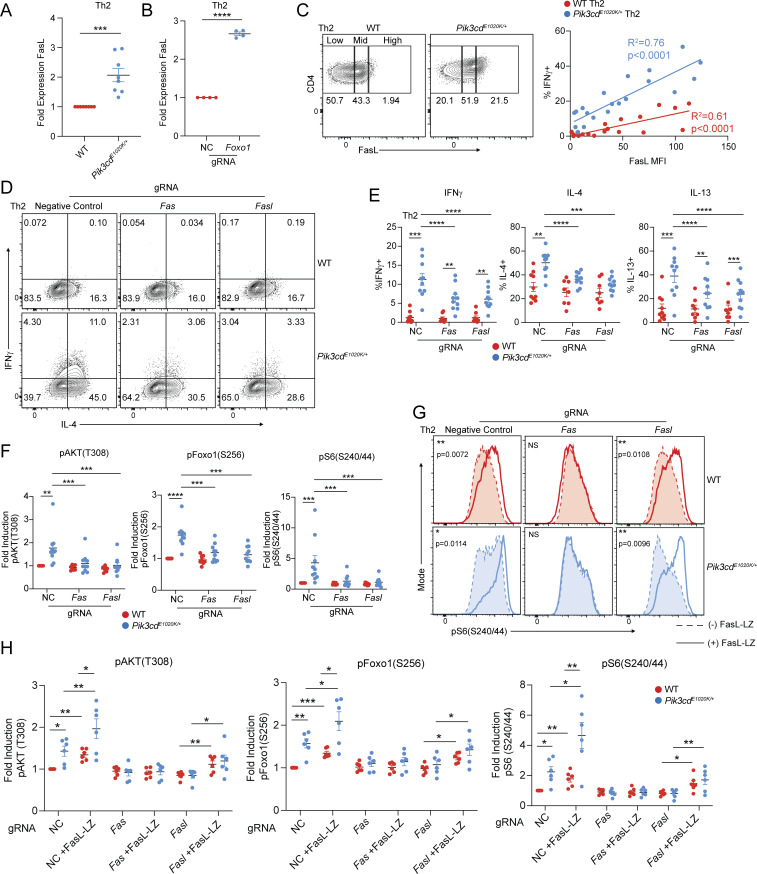
**Fas-FasL signaling potentiates T cell activation, exacerbating CD4** ^**+**^ **T cell dysregulation in the presence of activated PI3Kδ. (A)** Fold surface FasL expression (MFI normalized to WT) on Th2-polarized live CD4^+^ T cells, measured by flow cytometry (gated on live CD4^+^). *n* = 8 for each group, from eight independent experiments. **(B)** Fold surface FasL expression (MFI normalized to NC) on NC and *Foxo1* gRNA-targeted naïve CD4 T cells cultured under Th2-polarizing conditions. *n* = 4 for each group, from four independent experiments. **(C)** Left: Th2 polarized cells (live CD4^+^) from WT and *Pik3cd*^*E1020K/+*^ animals were gated as FasL^low^, FasL^mid^, and FasL^high^. Right: Linear regressions comparing FasL MFIs in each gate with percentages of IFNγ^+^ cells from the indicated groups. Correlation R^2^ and P values are indicated. *n* = 7 for each group, from seven independent experiments. **(D–F)** Naïve CD4 T cells from WT and *Pik3cd*^*E1020K/+*^ mice were nucleofected with Cas9-gRNA complexes containing NC, or *Fas*- or *Fasl*-targeting gRNAs and polarized under Th2 conditions. *n* = 8–10 for each group, from 8 to 10 independent experiments. **(D)** Representative flow cytometry plots showing IFNγ and IL-4 expression in live CD4^+^ T cells. **(E)** Percentages of IFNγ^+^ (left), IL-4^+^ (middle), and IL-13^+^ (right) in Th2-polarized live CD4^+^ T cells. **(F)** Fold induction of pAKT(T308) (left), pFoxo1(S256) (middle), and pS6(S240/44) (right) in Th2-polarized live CD4 T cells, measured by flow cytometry. For all readouts, fold induction was calculated by normalizing MFIs to NC WT cells. **(G and H)** NC, *Fas*, and *Fasl* gRNA-treated naïve CD4 T cells underwent Th2 polarization in the presence or absence of recombinant multimeric FasL (FasL-LZ), and phosphorylation of AKT(T308), Foxo1(S256), and S6(S240/44) was analyzed by flow cytometry. *n* = 6 for each group, from six independent experiments. **(G)** Representative flow cytometry histograms showing pS6(S240/44) staining in Th2-polarized live CD4^+^ T cells. Dashed lines represent cells cultured without FasL-LZ; solid lines show cells cultured with FasL-LZ. **(H)** Fold induction of pAKT(T308), pFoxo1(S256), and pS6(S240/44) in Th2-polarized (–/+ FasL-LZ) live CD4^+^ T cells, measured by flow cytometry. For all readouts, fold induction was calculated by normalizing MFIs to NC WT cells. Statistical comparisons were made using ratio paired *t* tests, unless otherwise indicated. *P < 0.05, **P < 0.01, ***P < 0.001, ****P < 0.0001. NC, negative control.

To provide additional insight into phenotypes of *Pik3cd*^*E1020K/+*^ Th2 cells, we explored potential roles of specific Foxo1-regulated genes. Among these, we noted that *Fasl* was significantly elevated in *Pik3cd*^*E1020K/+*^ CD4 T cells (Fig. 5 D); increased expression of FasL was confirmed by surface staining and flow cytometry of *Pik3cd*^*E1020K/+*^ Th2 cells (Fig. 7 A and Fig. S5 A). *Foxo1* gRNA-targeted Th2 cells also showed elevated surface FasL expression compared with negative control gRNA-treated cells (Fig. 7 B), whereas αIL-2–treated *Pik3cd*^*E1020K/+*^ Th2 cells showed diminished FasL compared with control counterparts (Fig. S5 A). The expression of FasL in *Pik3cd*^*E1020K/+*^ Th2 cells therefore paralleled the patterns observed for IFNγ.

FasL stimulates Fas, a TNF receptor family member that is best known for its role in modulating apoptosis. However, there is also evidence that nonapoptotic effects of Fas contribute to T cell activation and function (Akimzhanov et al., 2010; Cruz et al., 2016; Klebanoff et al., 2016; Paulsen et al., 2011). To probe the relationship between FasL and phenotypes of *Pik3cd*^*E1020K/+*^ cells, we gated FasL surface expression on Th2 cells as FasL^low^, FasL^mid^, or FasL^high^ and evaluated expression of IFNγ (Fig. 7 C). While WT cells were primarily FasL^low^ and FasL^mid^, *Pik3cd*^*E1020K/+*^ cells were enriched for FasL^mid^ and FasL^high^ cells (Fig. 7 C and Fig. S5 B). In both WT and *Pik3cd*^*E1020K/+*^ Th2-polarized cells, production of IFNγ positively correlated with the increasing expression of FasL (Fig. 7 C), although *Pik3cd*^*E1020K/+*^ cells expressed higher percentages of IFNγ that increased more rapidly as levels of FasL increased (Fig. 7 C and Fig. S5 C). FasL expression also correlated with cell death, and *Pik3cd*^*E1020K/+*^ Th2 cells also showed significantly elevated percentages of dead cells compared with WT, but WT cells were more sensitive to cell death at high levels of FasL (Fig. S5, D and E).

The correlation of IFNγ expression with increased FasL could reflect the activation status of *Pik3cd*^*E1020K/+*^ cells or, alternatively, indicate a direct role of FasL/Fas in these phenotypes. To determine whether FasL-Fas signaling directly affects Th2 differentiation, we used CRISPR/Cas9 gene targeting to ablate either *Fas* or *Fasl* in naïve CD4 T cells prior to Th2 polarization *in vitro* (Fig. S5, F and G). WT cells treated with *Fas*- or *Fasl*-targeting gRNAs showed no changes in IL-4, IL-13, or IFNγ production (Fig. 7, D and E). However, *Fas-* or *Fasl*-targeted *Pik3cd*^*E1020K/+*^ cells showed reduced percentages of both Th2 cytokine–producing cells (IL-4, IL-13) and, notably, significantly decreased IFNγ-expressing cells under Th2 conditions (Fig. 7, D and E). Thus, Fas-FasL signaling modulates aberrant cytokine production in *Pik3cd*^*E1020K/+*^ Th2 cells.

We next evaluated the consequences of Fas-FasL ablation on *Pik3cd*^*E1020K/+*^ cell signaling. Surprisingly, we observed significantly diminished pAKT(T308), a proximal readout of PI3Kδ activity, in *Fas*- or *Fasl*-targeted *Pik3cd*^*E1020K/+*^ Th2 cells compared with control, with activation resembling control WT cells (Fig. 7 F and Fig. S5 H). pFoxo1(S256) and pS6(S240/44) were similarly reduced by *Fas* or *Fasl* ablation in *Pik3cd*^*E1020K/+*^ Th2 cells (Fig. 7, F–H; and Fig. S5 H), as was phosphorylation of p65/RelA (Fig. S5 I), a component of NF-κB that has previously been found to be activated by noncanonical Fas signaling (Kreuz et al., 2004; Magri et al., 2024; Packham et al., 1997).

Since FasL contains intracellular motifs that might contribute to signaling, these phenotypes could result from either effects of FasL stimulation of Fas or signaling through the FasL intracellular domain (Malarkannan, 2020; Sun et al., 2006). To address this question, we used a recombinant FasL construct with an engineered leucine zipper domain that facilitates multimerization (FasL-LZ) to mimic effects of increased FasL. Both WT and *Pik3cd*^*E1020K/+*^ Th2 cells differentiated in the presence of FasL-LZ showed significantly increased pS6(S240/44) compared with control Th2 cells (Fig. 7, G and H), suggesting these effects resulted from stimulation of Fas. Increased signaling was dependent on the presence of Fas; Fas-deficient cells were completely resistant to the effects of FasL, whereas FasL-deficient cells retained the ability to respond to the inclusion of FasL-LZ in culture (Fig. 7, G and H). Identical trends were observed for pFoxo1(S256) and pAKT(T308), with FasL-LZ stimulation further exacerbating activation of *Pik3cd*^*E1020K/+*^ Th2 cells (Fig. 7 H). Thus, engagement of Fas amplified signaling pathways in *Pik3cd*^*E1020K/+*^ CD4 T cells.

The activation of downstream signaling by exogenous FasL-LZ suggested these pathways are activated in *trans* or a non–cell-autonomous manner. Nonetheless, the percentage of cells expressing IFNγ was proportional to the amount of FasL expressed on the same cell (Fig. 7 C). Furthermore, coculture experiments using equal numbers of WT and *Pik3cd*^*E1020K/+*^ naïve CD4 T cells under Th2-polarizing conditions revealed only minor increases in IFNγ expression in WT cells, while *Pik3cd*^*E1020K/+*^ cells maintained elevated frequencies of IFNγ^+^ cells (Fig. S5 J). Together, these data argue that the costimulatory effects of FasL on *Pik3cd*^*E1020K*^ cells can act in *cis* or *trans*, but are primarily cell intrinsic.

### Nonapoptotic Fas signaling occurs in the absence of FADD

**Figure 8.**
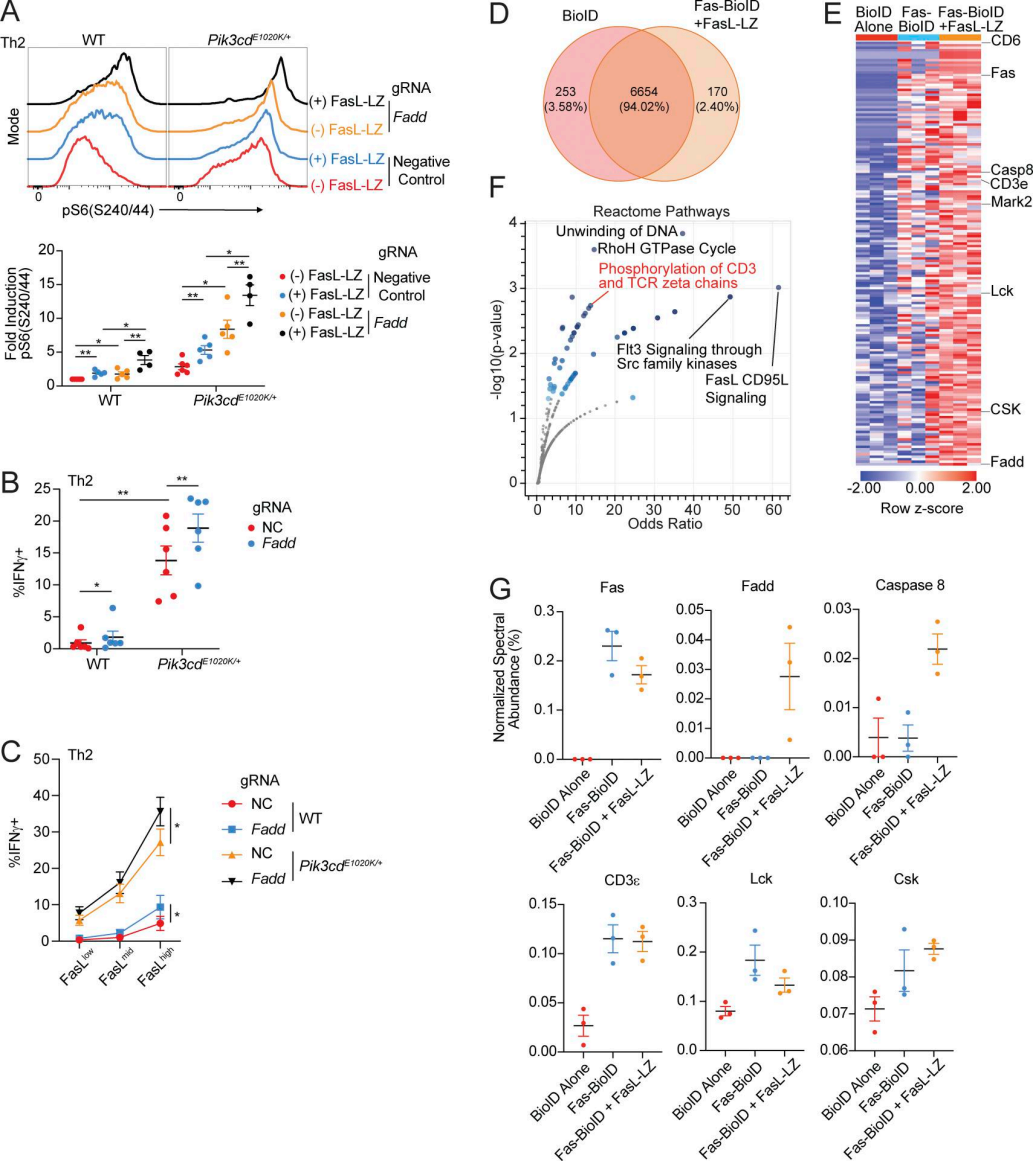
**Fas-induced T cell activation occurs in the absence of FADD. (A)** Naïve CD4 T cells from WT and *Pik3cd*^*E1020K/+*^ mice were nucleofected with Cas9-gRNA complexes containing NC or *Fadd*-targeting gRNAs and polarized under Th2 conditions in the presence or absence of FasL-LZ (25 ng/ml). Top: Representative flow cytometry histograms showing pS6(S240/44) staining in live CD4^+^ cells. Bottom: Fold induction of pS6(S240/44) in Th2-polarized (−/+ FasL-LZ) live CD4^+^ T cells. Fold induction was calculated by normalizing MFIs to NC WT cells. *n* = 4–5 for each group, from four to five independent experiments. **(B and C)** Naïve CD4 T cells from WT and *Pik3cd*^*E1020K/+*^ mice were nucleofected with Cas9-gRNA complexes containing NC or *Fadd*-targeting gRNAs and polarized under Th2 conditions. *n* = 6 for each group, from six independent experiments. **(B)** Frequencies of IFNγ^+^ Th2-polarized cells. **(C)** Th2-polarized cells were gated as FasL^low^, FasL^mid^, and FasL^high^. Frequencies of IFNγ^+^ cells were measured in the indicated groups. **(D–G)** Fas was tagged with BioID2 on its intracellular C terminus (Fas-BioID) and stably expressed in Fas-deficient Jurkat cells. Fas-BioID Jurkat cells were cultured in the presence or absence of recombinant multimeric FasL (FasL-LZ). Jurkat cells expressing BioID alone were used as a control. **(D)** Venn diagram showing proteins identified following mass spectrometry analysis of biotinylated proteins (streptavidin pull-down) that were common between or specific to BioID-alone Jurkat cells versus Fas-BioID + FasL-LZ (P < 0.05, *n* = 3 for all groups). **(E)** Heatmap (row z-score) showing % normalized spectral abundance of proteins specifically upregulated in Fas-BioID ± FasL-LZ Jurkat cells relative to BioID-alone Jurkat cells. **(F)** Pathway enrichment of Reactome gene sets was performed using Enrichr (Xie et al., 2021), with significantly enriched gene sets colored in blue; proteins specifically upregulated in Fas-BioID + FasL-LZ Jurkat cells relative to BioID-alone Jurkat cells were used as input for pathway enrichment. **(G)** Normalized spectral abundance (%) of the indicated proteins in BioID-alone, Fas-BioID, and Fas-BioID + FasL-LZ Jurkat cells, measured by mass spectrometry. Statistical comparisons were made using ratio paired *t* tests (G). *P < 0.05, **P < 0.01. NC, negative control.

Fas engagement by multimerized FasL causes formation of the death-inducing signaling complex (DISC), which results in the induction of apoptosis. To understand mechanisms contributing to nonapoptotic signaling, we used CRISPR/Cas9 to target *Fadd* (Fig. S5 K), the immediate downstream adapter that associates with Fas to generate the DISC, in naïve CD4 T cells that were then differentiated under Th2 conditions. Deletion of FADD did not prevent the induction of pS6(S240/44) by FasL-LZ in either WT and *Pik3cd*^*E1020K/+*^ Th2 cells (Fig. 8 A). Furthermore, FADD deficiency alone increased pS6(S240/44) induction in both WT and *Pik3cd*^*E1020K/+*^ Th2 cells, suggesting that loss of FADD biased Fas activity toward nonapoptotic signaling. Indeed, *Fadd*-targeted Th2-polarized cells showed significantly elevated IFNγ production compared with negative control counterparts (Fig. 8 B), and this correlated with increasing expression of FasL (Fig. 8 C). Together, these results suggest that FasL-driven regulation of IFNγ production occurs via a pathway independent of DISC formation.

### Fas costimulates TCR signaling

To investigate mechanisms behind Fas-induced amplification of PI3K pathways, we fused the cytoplasmic tail of Fas to a biotin ligase (BioID2), which we expressed in a Jurkat cell line that lacks endogenous Fas. BioID screening allows for the identification of potential protein–protein interactions through the transfer of biotin groups from a tagged bait protein to interacting partners, which can be isolated and identified by mass spectrometry. Using this approach, we analyzed 7,077 total proteins, with FasL-LZ stimulated Fas-BioID cells showing specific enrichment of 170 proteins relative to Jurkat cells expressing BioID alone (Fig. 8 D and Table S4). Among these Fas-BioID–specific proteins, we identified known Fas-associated proteins including Fas itself, FADD, and caspase-8; these latter two were only labeled upon FasL-LZ stimulation, confirming the fidelity of our Fas-BioID approach (Fig. 8, E–G). However, in addition to known Fas-associated proteins, we also observed significant interactions with multiple TCR signaling molecules including CD3ε and Lck (Fig. 8, E–G). Pathway enrichment analysis of Fas-BioID+FasL-LZ–specific proteins identified phosphorylation of CD3 and TCRζ chains among the top enriched pathways (Fig. 8 F).

**Figure 9.**
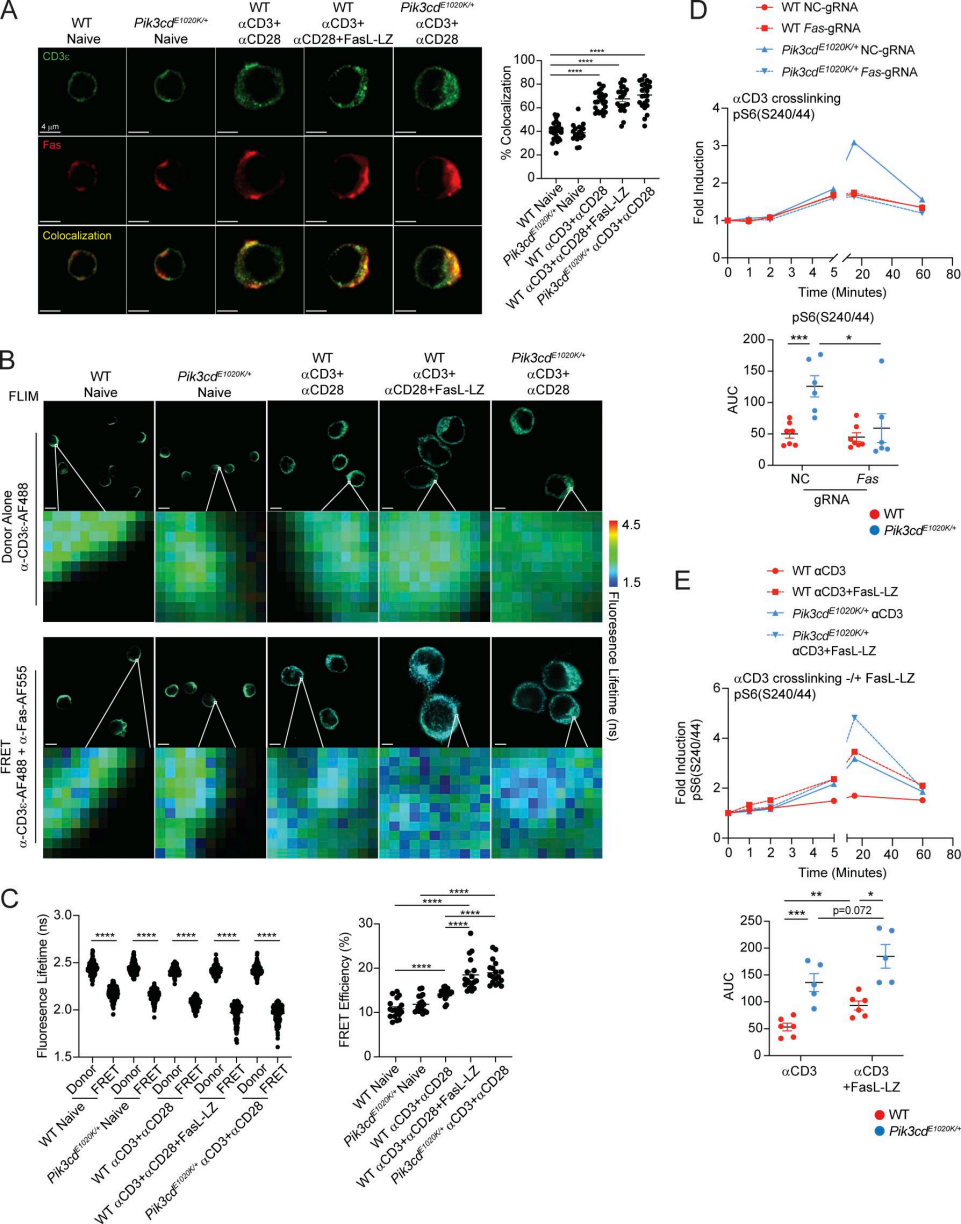
**Fas interacts with the TCR complex and costimulates TCR signaling. (A)** Confocal imaging of CD3ε and Fas in CD4 T cells. Naïve and stimulated (αCD3+αCD28 or αCD3+αCD28+FasL-LZ) CD4 T cells from the indicated mice were stained with αCD3ε-AF488 (green) and αFas-AF555 (red), and colocalization (yellow) was measured. Data are representative of three individual experiments, *n* = 19–24 unique images for each group. Left: Representative images of CD3ε and Fas costaining from the indicated groups. Right: Quantification of % colocalization between CD3ε and Fas in the indicated groups. **(B and C)** FLIM-FRET microscopy of CD3ε-Fas interactions in CD4 T cells. Naïve and stimulated (αCD3+αCD28 or αCD3+αCD28+FasL-LZ) CD4 T cells from the indicated mice were stained with αCD3ε-AF488 alone (donor control) or costained with αCD3ε-AF488 (donor) and αFas-AF555 (acceptor), and FL was measured. Results are representative of three independent experiments. **(B)** Representative images from the indicated groups. Scale bars in images indicate 4 μm. **(C)** Left: FL (ns) measurements of individual pixels from ROIs on cells from indicated groups. Right: FRET efficiency (%) within ROI from individual cells from the indicated groups. *n* = 18 for each group. **(D)** Naïve CD4 T cells from WT and *Pik3cd*^*E1020K/+*^ mice were nucleofected with Cas9-gRNA complexes containing NC or *Fas*-targeting gRNAs and stimulated with αCD3/CD28 in the presence of hIL-2 for 72 h. Cells were rested in serum-free media, treated with αCD3 (1 μg/ml) (0, 1, 2, 5, 15, 60 min), and were analyzed by flow cytometry. *n* = 6–7 for each group, from six to seven independent experiments. Top: Fold induction of pS6(S240/44) over time. Fold induction was calculated using pS6(S240/44) MFIs normalized to the 0 time point of the corresponding sample. Bottom: AUC quantification of pS6(S240/44) time courses for the indicated groups. **(E)** Naïve CD4 T cells from WT and *Pik3cd*^*E1020K/+*^ mice underwent αCD3/CD28 stimulation in the presence of hIL-2 for 72 h. Cells were subsequently rested in serum-free media, treated with αCD3 (1 μg/ml) in the presence or absence of FasL-LZ over a time course (0, 1, 2, 5, 15, 60 min), and analyzed by flow cytometry. *n* = 6–7 for each group, from six to seven independent experiments. Top: Fold induction of pS6(S240/44) over time for the indicated groups. Fold induction was calculated using pS6(S240/44) MFIs normalized to the 0 time point of the corresponding sample. Bottom: AUC quantification of pS6(S240/44) time courses. Statistical comparisons were made using ratio paired *t* tests (D and E) and unpaired *t* tests (A and C). *P < 0.05, **P < 0.01, ***P < 0.001, ****P < 0.0001. AUC, area under the curve; NC, negative control; ROIs, regions of interest.

To evaluate potential interactions between Fas and CD3ε in primary mouse T cells, we performed confocal imaging of Fas- and CD3ε-stained naïve and activated CD4 T cells from WT and *Pik3cd*^*E1020K/+*^ mice (Fig. 9 A). While we observed moderate colocalization of Fas and CD3ε in naïve CD4 T cells, activated CD4 T cells showed significantly elevated percentages of colocalization relative to naïve cells. To examine molecular interactions between Fas and CD3ε, we performed fluorescence resonance energy transfer (FRET) studies using fluorescence lifetime imaging microscopy (FLIM), with αCD3ε-AF488 as donor and αFas-AF555 as acceptor (Fig. 9, B and C). Naïve and stimulated CD4 T cells stained with αCD3ε-AF488 alone showed an average fluorescence lifetime (FL) of ∼2.4 ns, consistent with the normal decay of AF488. Cells costained with αCD3ε-AF488 and αFas-AF555 showed significant decreases in FL, indicative of protein–protein interactions and FRET (Fig. 9, B and C). However, WT cells stimulated with αCD3+αCD28 showed significantly increased FRET efficiency compared with naïve counterparts, suggesting that T cell activation promotes CD3ε-Fas interactions (Fig. 9 C, right panel). Moreover, αCD3+αCD28+FasL-LZ–stimulated WT CD4 T cells or αCD3+αCD28–stimulated *Pik3cd*^*E1020K/+*^ CD4 T cells, which express high levels of FasL, showed significantly higher FRET efficiency than WT αCD3+αCD28-stimulated cells (Fig. 9 C). Together, these results provide evidence that both TCR and Fas engagement increase the proximity of Fas and CD3ε during activation.

To evaluate whether Fas directly influences TCR signaling, we activated control and *Fas*-targeted CD4 T cells for 3 days, then rested cells in serum-free media without cytokines, followed by acute αCD3 crosslinking. Using pS6(S240/44) as a readout of downstream TCR signaling, we confirmed significantly elevated phosphorylation in *Pik3cd*^*E1020K/+*^ cells relative to WT cells (Fig. 9 D). However, Fas-deficient *Pik3cd*^*E1020K/+*^ CD4 T cells displayed impaired induction of pS6(S240/44) in response to αCD3 crosslinking (Fig. 9 D). Additionally, Fas-deficient *Pik3cd*^*E1020K/+*^ CD4 T cells also showed diminished pAKT(T308) and pERK(T202/04) responses compared with control cells (Fig. S5, L–M), confirming broad effects of the loss of Fas engagement on multiple T cell signaling pathways in activated PI3Kδ CD4 T cells.

**Figure 10.**
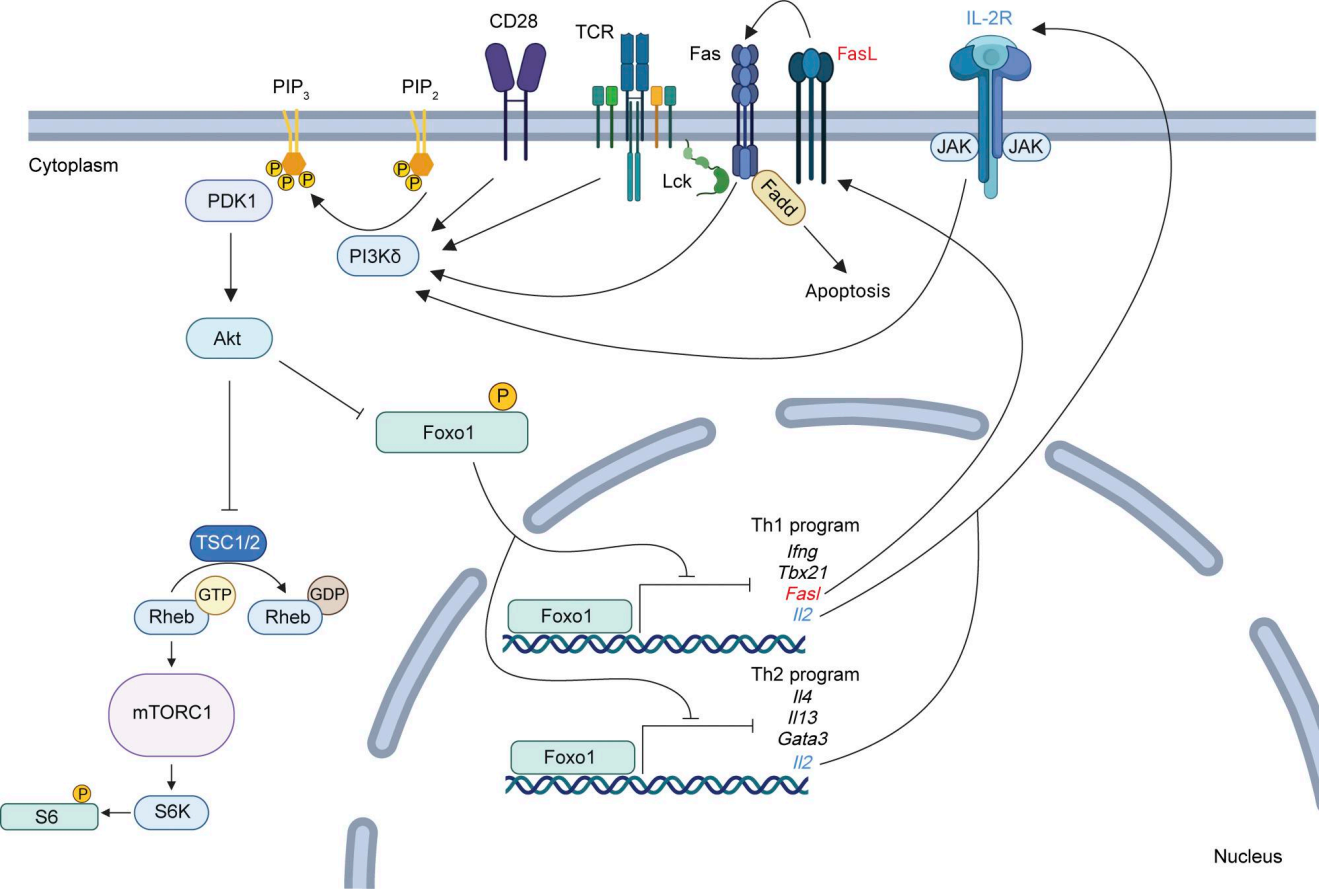
**PI3Kδ regulates CD4** ^**+**^ **T cell differentiation through integration of TCR, IL-2 receptor, and Fas signaling, driving Foxo1 inactivation and transcriptional reprogramming.**

Decreased TCR signaling was not observed in Fas-deficient WT CD4 T cells (Fig. 9 D and Fig. S5, L and M), likely due to their low expression of FasL (Fig. 7 A). To probe Fas-mediated regulation of TCR signaling in WT cells, we again activated CD4 T cells for 3 days, rested cells in serum-free media, and acutely crosslinked CD3 in the presence or absence of recombinant FasL-LZ (Fig. 9 E and Fig. S5, N and O). We observed significantly elevated S6 phosphorylation in WT CD4 T cells acutely stimulated through the TCR in the presence of FasL-LZ compared with control TCR-stimulated cells (Fig. 9 E). Indeed, WT cells stimulated with αCD3 in the presence of FasL-LZ showed similar amplification of pS6(S240/44) as *Pik3cd*^*E1020K/+*^ cells stimulated with αCD3 alone. Similarly, induction of pAKT(T308) and pERK(T202/04) was increased by FasL-LZ costimulation of WT cells (Fig. S5, N and O), again revealing broad downstream effects of Fas engagement. Moreover, FasL-LZ costimulation further elevated pS6(S240/44), pAKT(T308), and pERK(T202/04) in *Pik3cd*^*E1020K/+*^ CD4 cells (Fig. 9 E and Fig. S5, N and O). Together, these results suggest that activated PI3Kδ promotes T cell activation via a Foxo1-FasL-Fas amplification loop that potentiates TCR signaling and induces IFNγ and type I inflammatory gene expression, disrupting Th2 lineage restriction (Fig. 10).

## Discussion

We have uncovered a unique PI3Kδ signaling circuit that enforces transcriptional and epigenetic programs enforcing Th2 lineage fidelity. Activation of PI3Kδ downstream of TCR drives both inactivation of Foxo1 and elevated IL-2 signaling, including increased production of IL-2 and exaggerated IL-2–induced Foxo1 inactivation, enforcing a positive amplification loop that drives IFNγ expression and prevents Th2 lineage restriction. We have further identified Foxo1-repressed Fas-FasL signaling as a novel mechanism contributing to T cell functional outcomes, via amplification of signaling from the TCR, associated with major changes in the cellular transcriptional and epigenetic landscape. Together, this dynamic signaling loop generates a self-reinforcing signaling circuit that results in robust CD4 T cell activation and differentiation that may contribute to the effects of PI3Kδ hyperactivation in APDS.

APDS patients show numerous pathologies in the respiratory tract, including frequent recurrent respiratory infections and bronchiectasis (Condliffe and Chandra, 2018). Nonetheless, despite reports of increased incidence of asthma (Bier et al., 2019; Tangye et al., 2019), it remains unclear whether patients show a classical eosinophilic asthma versus other lung pathologies. *In vitro*, CD4 T cells from APDS patients and *Pik3cd*^*E1020K/+*^ mice show increased IL-4 and IL-13 production under Th2 conditions (Bier et al., 2019; Preite et al., 2019), yet how this response proceeds in patients *in vivo* is unclear. Indeed, despite elevated production of both Th1 and Th2 cytokines under polarizing conditions *in vitro* (Bier et al., 2019; Jia et al., 2021; Preite et al., 2019), we found that PI3Kδ hyperactivation drives a highly dysregulated response to HDM in lung tissue, with increased infiltration of CD4 T cells and neutrophils, yet impaired Th2 responses and enhanced IFNγ signatures. These somewhat contradictory findings are likely due to the effects of inappropriate production of IFNγ by CD4 T cells expressing activated PI3Kδ and the strong Th1-polarizing effects of IFNγ seen in scenarios of mixed Th1 and Th2 responses, particularly in the lung (Cautivo et al., 2022; Erb et al., 1998; Gavett et al., 1995; Marsland et al., 2004); such effects may not be apparent under Th2 conditions *in vitro* in the presence of high amounts of exogenous IL-4. Indeed, previous work has shown elevated Tfh-like and total CD4^+^ cells from APDS patients expressing CXCR3, a marker of IFNγ-producing cells (Bier et al., 2019; Jia et al., 2021; Preite et al., 2018); APDS patients also have elevated serum IFNγ and CXCL10, an IFNγ-induced chemokine, both of which respond to leniolisib, a specific PI3Kδ inhibitor (Rao et al., 2017). It is of note that PI3Kδ inhibitors have been examined in certain lung diseases such as chronic obstructive pulmonary disease; whether their use may be beneficial in the context of neutrophilic asthma may warrant closer examination (Moradi et al., 2021; Yoo et al., 2017).

The role of Foxo1 as a transcriptional repressor has been described in various cell types; our data now demonstrate the interplay between PI3Kδ, IL-2, and Foxo1 in shaping CD4 lineage specification. In addition to uncovering a role of Foxo1 in repressing Th2 lineage cytokines IL-4 and IL-13, we further found that loss of Foxo1 mediates the induction of the Th1 program under Th2-inducing conditions, highlighting the requirement of balanced Foxo1 activity to ensure Th2 lineage determination. In addition, we show that Foxo1 repression results in elevated production of IL-2, driving further inactivation of Foxo1 and establishing a signaling circuit that permits simultaneous adoption of multiple CD4 T cell lineages in *Pik3cd*^*E1020K/+*^ cells. While the involvement of PI3K downstream of IL-2 has been controversial (Ross and Cantrell, 2018), we see evidence for this amplification circuit in the context of activated PI3Kδ, both in CD4^+^ and in CD8^+^ T cells (Cannons et al., 2021). In *Pik3cd*^*E1020K/+*^ CD8 T cells, elevated IL-2 responses drive enhanced effector differentiation at the expense of memory-like cell formation, accompanied by increased FasL-driven cell death (Cannons et al., 2021); however, in CD4 T cells this signaling circuit drives a loss of Th2 lineage restriction via a Foxo1-driven, Fas-mediated amplification of TCR signaling. Elegant work by DiToro et al. has demonstrated that IL-2–producing CD4 T cells and IL-2–responding CD4 T cells are distinct populations with unique patterns of differentiation (DiToro et al., 2018). In the context of activated PI3Kδ, we speculate that this paradigm may contribute to altered responses, with elevated frequencies of IL-2–producing cells providing hyperresponsive IL-2–responding cells with cytokine signals that facilitate dysregulated effector differentiation. Of note, PI3K-Akt signaling has also been shown to regulate GATA3 activity via phosphorylation by Akt1, leading to a loss of Tbet repression and the induction of IFNγ expression in Th2 cells (Hosokawa et al., 2016). Together, these results suggest there may be multiple mechanisms by which activated PI3Kδ conspires to alter Th2 lineage stability.

Multiple PI3K-mediated pathways contribute to phenotypes associated with APDS (Angulo et al., 2013; Jia et al., 2021; Johansen et al., 2022; Lucas et al., 2014); our data now demonstrate that nonapoptotic Fas-FasL signaling serves as a critical input exacerbating activation of *Pik3cd*^*E1020K/+*^ CD4 T cells. We had previously seen increased FasL led to increased cell death in CD8 T cells from activated PI3Kδ mice (Cannons et al., 2021); we now find that aberrant FasL expression exacerbates PI3Kδ hyperactivation and causes a loss of Th2 lineage restriction in CD4 T cells. The observation that Fas stimulation of WT CD4 T cells using recombinant FasL-LZ promoted amplification of PI3K and ERK signaling downstream of the TCR suggests this circuit can be part of normal T cell activation. BioID screening and FLIM-FRET imaging revealed intimate proximity between TCR components and Fas, providing a basis for the ability of Fas to potentiate TCR signaling, which appears to be distinct from its role in cell death via DISC formation nucleated by FADD. Our data further indicate that FADD restrains nonapoptotic Fas signaling, suggesting that failure to assemble the DISC may help dictate signaling outcomes downstream of Fas. Previous work has described cooperativity between Fas and TCR components in regulating both proximal and distal TCR signaling events (Akimzhanov et al., 2010; Paulsen et al., 2011; Tauzin et al., 2011); other evidence points toward a role of Fas in promoting acquisition of effector phenotypes in T cells (Klebanoff et al., 2016); these include roles of IFNγ-mediated resolution of an allergic asthma model (Tong et al., 2006; Tong et al., 2010), and in potentiating the activation of naïve T cells (Cruz et al., 2016). Furthermore, this amplification loop may have broad implications for other outcomes associated with PI3K activation, where nonapoptotic Fas signaling regulates a range of cellular processes in a variety of cell types, including glioblastoma, where it contributes to cell migration and invasion (Corsini et al., 2009; Gulbins et al., 1998; Kleber et al., 2008; Magri et al., 2024; Meyer Zu Horste et al., 2018; Peter et al., 2007; Staniek et al., 2024; Strauss et al., 2009; Tauzin et al., 2011; Yi et al., 2025). However, whether nonapoptotic Fas-FasL signaling is initiated by FasL in *cis* or *trans* is less clear; stimulation of CD4 T cells using recombinant FasL-LZ was able to potentiate nonapoptotic Fas signaling, indicating that *trans* FasL can trigger this pathway. Nonetheless, increased production of IFNγ correlated with levels of FasL expressed by individual CD4 T cells. Moreover, coculture experiments of WT and *Pik3cd*^*E1020K/+*^ CD4 T cells under Th2-polarizing conditions showed only minor elevations in cytokine production in WT CD4 T cells compared with *Pik3cd*^*E1020K/+*^ CD4 T cells in the same culture, suggesting that FasL plays an important role in *cis*. Future studies to elucidate mechanisms through which nonapoptotic Fas-FasL signaling is initiated will be of interest for understanding these signaling choices.

Our findings of dysregulated epigenetic remodeling driven by hyperactivated PI3Kδ also highlight an unappreciated role of this pathway in influencing chromatin organization during CD4 T cell differentiation. The increased accessibility of Th1-promoting motifs like Tbet, Runx, and IRF, and a loss of accessibility of Th2-associated motifs such as GATA3 and STAT6 in *Pik3cd*^*E1020K/+*^ CD4 Th2 cells are consistent with the loss of Th2 lineage restriction. However, more surprising was the attenuation of CTCF motif accessibility and CTCF-DNA binding in the context of PI3Kδ hyperactivation, highlighting the highly dysregulated nature of activated PI3Kδ. Nonetheless, deletion of Foxo1 did not affect CTCF expression, despite phenocopying Th2 differentiation of activated PI3Kδ cells, suggesting that CTCF is regulated by other PI3K-dependent but Foxo1-independent mechanism(s). It is of interest that partial loss of CTCF has been shown to globally increase variation in gene expression (Ren et al., 2017), which may contribute to variation in phenotypes observed with activated PI3Kδ. How PI3Kδ and CTCF cooperate to regulate T cell function warrants further examination.

Integration of PI3Kδ signaling with the transcriptional and epigenetic networks that orchestrate CD4 T cell differentiation requires a delicate balance of signals to ensure both robust T cell activation and appropriate differentiation. Excessive signaling through PI3Kδ disrupts this balance, causing a downstream cascade of cellular dysregulation at multiple levels. The ability of PI3Kδ to amplify its own activation through linking IL-2, Foxo1, and Fas-FasL signaling demonstrates a hard-wired mechanism for sustaining T cell activation during the acquisition of effector function. However, rewiring of this signaling circuit by activated PI3Kδ incites inappropriate amplification of signaling responses that cause T cell dysregulation and a loss of T cell lineage identity that may contribute to increased inflammation in multiple scenarios. How this contributes to phenotypes associated with altered PI3Kδ activity in immunodeficiencies and other diseases remains an important question.

## Materials and methods

### Experimental model details

#### Mice

*Pik3cd* ^*E1020K/+*^ mice have been described previously and were backcrossed >10 times to C57BL/6J. For all experiments, age- and sex-matched mice between 8 and 12 wk of age were used. For adoptive transfer experiments, TCRα-deficient mice (B6.129S2-*Tcra*^*tm1Mom*^) were obtained from National Institute of Allergy and Infectious Diseases (NIAID)-Taconic contract facility and used as recipients. Mice were maintained and treated under specific pathogen-free conditions in accordance with the approved protocols of Animal Care and Use committees of National Human Genome Research Institute (protocol G98-3) and NIAID (protocols LPD-6, LHIM-4E, and LISB-22E) at the National Institutes of Health (NIH) (#A- 4149-01; Animal Welfare Assurance).

### Method details

#### Antibodies and flow cytometry

All flow cytometry antibodies were purchased from BD, BioLegend, Thermo Fisher Scientific, or Cell Signaling Technologies (Table S5). Surface antibody staining was performed in FACS buffer (PBS supplemented with 1% FBS and 1 mM EDTA) for 30 min at 4°C. For TF staining, cells were stained using the Foxp3 staining kit (fixation for 25 min at room temperature, intracellular staining for a minimum of 1 h at 4°C). For intracellular cytokine analysis, cells were restimulated with PMA (50 ng/ml) and ionomycin (500 ng/ml) in the presence of GolgiStop (1/2,000) at 37°C for 4 h. Cytokine staining was performed using fixation in 4% PFA (25 min at room temperature) followed by intracellular staining in 0.5% Triton X-100, 0.1% BSA in PBS (minimum of 1 h at 4°C). Staining of phospho-proteins was performed using fixation in 4% paraformaldehyde (PFA) (25 min at room temperature) followed by permeabilization using ice-cold 100% methanol (1 h at 4°C) and intracellular staining in 0.5% Triton X-100, 0.1% BSA in PBS (minimum of 1 h at 4°C). Cells were acquired using a Fortessa (BD) flow cytometer and analyzed using FlowJo software (version 10.8.1).

#### HDM-induced airway inflammation

HDM extract (*Dermatophagoides pteronyssinus*) was purchased from Greer Laboratories and is extensively tested for endotoxin contamination. Mice were anesthetized with isoflurane and treated intranasally with 30 μl of HDM extract over the course of 18 days. On days 0 and 7, mice were treated with 200 μg of HDM extract. On days 14, 16, and 18, mice were treated with 50 μg of HDM extract. Following the last sensitization, mice were euthanized on day 20 and lung tissue was harvested for analysis. Lung tissues were dissected, and the left lung was used to make tissue homogenates for Luminex analysis, postcaval lobe was fixed for histological analysis, and superior, middle, and inferior lobes were pooled for digestion and flow cytometry analysis. To prepare single-cell suspensions for flow cytometry, lung tissue was cut into small pieces and digested in 0.25 mg/ml of Liberase DL for 45 min at 37°C. Tissue was subsequently processed through a 70-μm filter and treated with ACK lysis buffer to remove red blood cells. Single-cell suspensions were reconstituted in complete media, counted, and used for flow cytometry analysis. For adoptive transfer experiments, naïve CD4 T cells were isolated from spleen and lymph nodes of WT or *Pik3cd*^*E1020K/+*^ mice using Miltenyi naïve CD4 T cell isolation kits and 1 × 10^6^ cells per mouse were injected intraperitoneally into TCRα KO mice. Following injection, mice were rested for 14 days before initiating the HDM protocol outlined above.

#### Histology

The postcaval lobe of the lung was fixed in 10% formalin (buffered, 7.2 pH) and embedded in paraffin. Embedded tissues were sectioned (5 mm thick), mounted on glass slides, and underwent H&E staining to examine immune cell profiles. All slides were digitized using an Aperio AT2 scanner (Leica Biosystems).

The quantification of eosinophils was based on images from 10 randomly selected fields of lung sections per animal, captured at 40× magnification. All counts were carried out using Aperio ImageScope software (Leica Biosystems). Eosinophils were identified by the bilobed shape of their nucleus and acidophilic cytoplasm, and using the cursor and program tools, they were marked and counted (results expressed in absolute numbers).

The assessment of iBALT formation in mouse lungs was carried out by morphometric analysis as previously described (Oliveira et al., 2024), and 10 images were captured at 10× magnification to cover the entire area of the histological lung section using Aperio ImageScope software (Leica Biosystems). iBALT was identified by similar architecture to conventional secondary lymphoid organs, being found in the perivascular space surrounding large blood vessels and along the lung’s airways. The iBALT area was quantified using QuPath version 0.4.3 software (Bankhead et al., 2017) with results expressed as μm^2^.

The total mucosal area of the bronchi and bronchioles were obtained by morphometric analysis. All bronchial and bronchial mucosal areas were visualized by 10× magnification and captured using Aperio ImageScope software (Leica Biosystems) and then were manually measured to obtain the total area in µm^2^, using QuPath version 0.4.3 software. Subsequently, the same sections (analyzed previously) were visualized by the 4× magnification, captured using Aperio ImageScope software (Leica Biosystems) and the length of each mucosa was manually measured in micrometers to obtain the actual proportion of the mucosal surface analyzed for each mouse. Then, the ratio of the total area (µm^2^) of mucosa of each animal was normalized to the smallest mucosal length to obtain a value representing the mucosal area (µm^2^) of each mouse.

#### Analysis of airway resistance

Mice were anesthetized by intraperitoneal injection of a ketamine (100 mg/kg) and xylazine (20 mg/kg) mixture. Mice subsequently underwent transtracheal intubation with a 20-gauge SURFLO Teflon intravenous catheter (Santa Cruz Animal Health). Mice were administered vecuronium bromide (1 mg/kg) and mechanically ventilated using a flexiVent respirator (SCIREQ). Respiratory system resistance (*R*_rs_) was measured after inhalation of PBS and increasing concentrations of methacholine.

#### Measurement of lung cytokines and chemokines in tissue homogenates

Following lung dissection, left lobes were isolated and added to an ice-cold mixture of 500 μl RIPA buffer supplemented with protease inhibitor. Using Bertin hard tissue grinding tubes, samples were homogenized for 2 cycles of 20 s at 5,500 rpm in a Precellys Evolution (Bertin). Homogenates were subsequently centrifuged at 8000 × *g* for 10 min to remove debris, and supernatants were collected. Cytokine and chemokines were measured in homogenates using a 32plex Luminex kit (Millipore) and a Bio-Plex 200 analyzer (Bio-Rad) per the manufacturer’s instructions.

#### Measurement of HDM-specific antibodies

Generation of HDM-specific antibodies was determined by ELISA. Plates (Immunolon 4HBX) were coated with 5 μg/ml HDM extract (Greer Laboratories), incubated overnight at 4°C, and blocked with 5% BSA in PBS. Samples were measured in duplicate with 50 μl of serum samples added to HDM-coated wells (IgG1 [1/500], IgE [1/1], IgG2a [1/500], IgG3 [1/500]) and incubated at room temperature for 1 h. Samples were washed with Tween/PBS and then probed with biotinylated rat anti-mouse IgG1, IgE IgG2a, or IgG3 (1/250) and incubated at room temperature for 1 h. Following washing, streptavidin-conjugated HRP was added to samples and incubated at room temperature for 30 min. Samples were washed and treated with 3, 3′,5,5′-tetramethylbenzidine, with 2N H_2_SO_4_ used to stop the reaction. Plates were analyzed using a multiplate reader (Molecular Devices) at absorbances of 450 and 550 nm.

#### scRNAseq

Lungs were isolated from naïve and HDM-treated WT and *Pik3cd*^*E1020K/+*^ animals (*n* = 3 for all groups), and single-cell suspensions were prepared as previously described. Live CD45^+^ cells were sorted using an Aria Fusion cell sorter (BD), and cells were labeled with unique hashtag antibodies (BioLegend). Labeled cells were pooled by group (WT naïve, *Pik3cd*^*E1020K/+*^ naïve, WT HDM, *Pik3cd*^*E1020K/+*^ HDM) with 1 × 10^4^ cells per donor (*n* = 3 per group) used as input. Chromium Single Cell 3′ version 2 libraries (10x Genomics) were generated following the standard manufacturer’s protocol and sequenced on one NovaSeq S2 and NovaSeq SP run at the Frederick National Laboratory for Cancer Research Sequencing Facility. The sequencing run was set up as a 28 cycles + 91 cycles nonsymmetric run. All samples had sequencing yields of >206 million reads per sample. Samples were demultiplexed, allowing one mismatch in the barcodes. Over 93.7% of bases in the barcode regions had Q30 or above, and at least 89.8% of bases in the RNA read had Q30 or above. More than 93.8% of bases in the UMI had Q30 or above. Data were analyzed using Cell Ranger version 6.0.2 (Zheng et al., 2017), following default parameters, mapping to the *Mus musculus* Genome Reference Consortium Mouse Build 38 (mm10, accession: GCA_000001635.2, https://www.ncbi.nlm.nih.gov/assembly/GCF_000001635.20/).

After alignment to mm10, followed by the quantification of RNA counts per cell, Seurat version 4.1.1 (Hao et al., 2021) was used to filter the data and perform downstream analyses. Each dataset was visualized to confirm there were not bimodal distributions with an excess of cells with multiple hashtags and to determine the best cutoffs for cells lacking unique features or potential doublets. Cells were retained with >200 unique features but <3,000, and with <5% of reads mapping to mitochondrial genes.

Following filtering, we integrated samples based on the variable features of each cell, and then clustered cells, following default clustering parameters, with a resolution of 0.25. Numbers of CD45^+^ lung cells identified for analysis are as follows: WT naïve (7,077), *Pik3cd*^*E1020K/+*^ naïve (5,533), WT HDM (2,111), *Pik3cd*^*E1020K/+*^ HDM (14,119). Cell types were identified in the integrated dataset using a combination of cluster-specific gene expression data (Table S1) and the ImmGen database in SingleR version 1.8.1 (Aran et al., 2019). Seurat version 4.1.1 was used to generate dimension plots showing clusters and cell types of these data, as well as to generate features plots of gene expression levels. To balance cell numbers for visualization in dimensional plots, data groups were downsampled to 2,000 cells per condition. Enriched functional gene sets were identified using ShinyGo (Ge et al., 2020) and Enricher (Xie et al., 2021), with DEGs for indicated comparisons used as input.

To examine the response to IFNγ (GO:0034341) gene set in all cell clusters identified, we used the AUCell R package (Aibar et al., 2017). Briefly, we converted our data into an expression matrix of read counts per cell and identified whether genes contained in response to IFNγ gene set were expressed in each cell. We then used AUCell to generate an enrichment score of response to IFNγ in all individual cells compared with all other genes. To visualize these results, we used Seurat to plot enrichment P values as a dimension plot and barplot. We used chi-squared tests to determine whether there was a significant difference between WT and *Pik3cd*^*E1020K/+*^ in the proportion of cells with highly significant (P < 0.0005) enrichment P values.

#### Intranasal *C. neoformans* infection

*C. neoformans* was cultured overnight in YPAD at 30°C from individual colonies. Following overnight culture, yeast were counted on a hemocytometer and diluted to 2 × 10^6^ cells per ml in saline. Mice were anesthetized with intraperitoneal ketamine/dexmedetomidine, and 25 μl yeast (5 × 10^4^ CFU) was then pipetted onto the nasal flares and taken up by aspiration. Mice were sacrificed 10 days after infection, and lung tissue was harvested and digested as previously described for flow cytometry analysis.

#### *In vitro* CD4 T cell polarization

Naïve CD4 T cells were isolated from spleen and lymph nodes of WT and *Pik3cd*^*E1020K/+*^ mice using Miltenyi mouse naïve CD4 T cell isolation kits. APCs were prepared by depleting T cells from splenocyte suspensions by labeling with CD4-FITC (1/200) and CD8a-FITC (1/200) and negatively selecting labeled cells using anti-FITC MicroBeads (Miltenyi). Naïve T cells (2 × 10^5^) and APCs (1 × 10^6^) were mixed at 1:5 ratio and were plated into wells of a 48-well plate in 1 ml of complete IMDM (10% FBS, penicillin/streptomycin, glutamine, and 2-ME). Naïve CD4 T cells were activated with anti-CD3ε (1 μg/ml) and anti-CD28 (3 μg/ml) in the presence of cytokines and blocking antibodies (Perry et al., 2025). For Th1 conditions, cells were activated in the presence of IL-12 (20 ng/ml) and anti-IL-4 (10 μg/ml). For Th2 conditions, cells were activated in the presence of IL-4 (40 ng/ml) and anti-IL-12 (20 μg/ml). Cells were cultured for 24–72 h at 37°C (5% CO_2_) and analyzed at various time points during differentiation.

#### Bulk RNAseq

Live naïve CD4 T cells (CD4^+^CD8α^−^TCRβ^+^CD25^−^CD44^−^CD62L^+^) were sorted from spleens of WT and *Pik3cd*^*E1020K/+*^ mice and were resuspended in 1 ml of TRIzol (*n* = 3 for each group). Th1- and Th2-polarized cells (−/+ αIL-2) were generated *in vitro* from WT and *Pik3cd*^*E1020K/+*^ cells (as described), and live CD4^+^ cells were sorted directly into TRIzol (1 ml final volume). Total RNA was isolated from samples using phenol–chloroform extraction with GlycoBlue as a coprecipitant. RNA was quantified using Qubit RNA high-sensitivity reagents, and 30 ng of total RNA was used as input for RNAseq library preparation. Libraries were prepared using the NEBNext poly(A)^+^ mRNA magnetic isolation module and NEBNext Ultra RNA Library Prep Kit. Libraries were sequenced using the NovaSeq platform (Illumina) and underwent paired-end sequencing to produce between 262 and 540 million 100-bp read pairs per sample, for a total of 13.8 billion read pairs.

Raw base calls were demultiplexed and converted into FastQ format with CASAVA 1.8.2. The raw data were processed using the OpenOmics/RNA-seek pipeline version 1.9.0 (https://github.com/OpenOmics/RNA-seek). Briefly, this pipeline assessed the quality of each sample using FastQC version 0.11.9 (Babraham Bioinformatics, 2025), Preseq version 2.0.3 (Daley and Smith, 2013), Picard tools version 2.17.11 (Broad Institute, 2025), FastQ Screen version 0.9.3 (Wingett and Andrews, 2018), Kraken2 version 2.0.8 (Wood et al., 2019), QualiMap (Garcia-Alcalde et al., 2012), and RSeQC version 2.6.4 (Wang et al., 2012). Adapters and low-quality sequences were trimmed using Cutadapt version 1.18 (Martin, 2011). The trimmed reads were aligned against the *Mus musculus* Genome Reference Consortium Mouse Build 38 (mm10, accession: GCA_000001635.2, https://www.ncbi.nlm.nih.gov/assembly/GCF_000001635.20/), using the splicing-aware aligner STAR version 2.7.6a (Dobin et al., 2013) in per-sample two-pass basic mode. Gene and transcript expression levels were estimated via RSEM version 1.3.3 (Li and Dewey, 2011).

Transcripts were normalized and DEGs called by quasi-likelihood F testing using edgeR. DEG call denotes >1.5-fold pairwise change and Benjamini–Hochberg adjusted P value <0.05. Reads per kilobase per million (RPKM) were compiled with edgeR. An offset value of 1 was added to all RPKM, and those failing to reach a value >2 RPKM in any genotype/condition were purged, as were microRNAs, snoRNAs, and scaRNAs. RPKM+1 was the input for principal component analysis (prcomp).

clusterprofiler was used for hypergeometric testing (HGT) and GSEA against the KEGG, GO, Reactome and Molecular Signatures databases, or custom Foxo1-activated and Foxo1-repressed gene sets (Table S2). For HGT, input DEG lists were generated based on the above pairwise criteria. For GSEA, unabridged transcriptomes were ranked by Log2FC. GSEA plots were rendered with enrichplot, heatmaps with Morpheus (https://software.broadinstitute.org/morpheus), and all other plots with ggplot2 or DataGraph (Visual Data Tools Inc.).

#### CRISPR/Cas9 gene targeting

Predesigned CRISPR RNAs (crRNAs) were purchased from IDT and were reconstituted at 100 μM (see table for sequence details). Trans-activating CRISPR RNA (tracrRNA) labeled with atto550 was purchased from IDT and reconstituted at 100 μM. gRNAs were generated by mixing crRNA and tracrRNA at a 1:1 ratio and duplexed by warming to 95°C for 5 min and were cooled at room temperature for 10 min before use. For each gene targeted, a mixture of three gRNAs was used to ensure efficient gene ablation. RNP complexes were formed by incubating gRNAs (3 × 1 μl) with 1 μl recombinant Cas9 (IDT) at room temperature for 5 min. Naïve CD4 T cells were isolated (as previously described), resuspended in 20 μl Lonza P3 nucleofection buffer, and mixed with RNP complexes. Naïve CD4 T cells underwent nucleofection using P3 Primary Cell 4D-Nucleofector X Kit S cuvettes and the DN100 program of a Lonza 4D-Nucleofector device. Following nucleofection, cells were washed and cultured in complete IMDM supplemented with 5 ng/ml IL-7. The IL-7 culture period ranged from 1 to 7 days, depending on the target gene of interest. Negative control crRNA #1 was purchased from IDT and used in all negative control conditions for CRISPR/Cas9 experiments. Detailed protocols describing this method have been previously published (Perry et al., 2025), and gRNA sequences are listed in Table S6.

#### Retroviral transduction

For retrovirus production, HEK293T cells were cotransfected with GFP-pMIGR, Foxo1-WT-pMIGR, or Foxo1-AAA-pMX (2 μg) along with pCL-Eco helper plasmid (1 μg). 48 h after transfection, cell supernatants were collected and incubated with 5× PEG-IT reagent (SBI) overnight at 4°C. Precipitated retrovirus was centrifuged at 1,500 × *g* for 30 min, and supernatants were discarded. Retrovirus was centrifuged again at 1,500 × *g* for 5 min, the supernatant was discarded, and concentrated retrovirus was reconstituted in complete IMDM. For retroviral transduction, naïve CD4 T cells were activated as described above. 18 h after activation, concentrated retrovirus (20 μl) was mixed with polybrene A (8 µg/ml final), added to 1 ml of Th2-polarized cells in a 48-well plate, and spinfected at 2,000 rpm for 1 h at 37°C. Transduced cells were subsequently cultured at 37°C for a total of 72 h, without changing differentiation media. Transduced cells were detected by flow cytometry using GFP expression.

#### Recombinant FasL-LZ

Recombinant FasL-LZ was engineered by fusing the extracellular domain of Fas ligand to a FLAG tag and an isoleucine zipper motif that facilitates self-oligomerization of the construct (Cruz et al., 2016). This construct was transfected into HEK293T cells, and cell supernatants were collected. Recombinant FasL-LZ was purified using magnetic beads conjugated to anti-FLAG (Sigma-Aldrich). Quantification of recombinant protein was determined by ELISA (R&D). For all mouse CD4 T cell experiments, FasL-LZ was used at a concentration of 25 ng/ml. For Jurkat BioID screening, FasL-LZ stimulation was performed at 10 ng/ml.

#### Fas-BioID screening

Human *Fas* coding sequencing tagged with BioID2 (#80899; Addgene) and P2A-EGFP at the C terminus was subcloned into the pMY retroviral backbone (#163361; Addgene) using the Gibson assembly. Retrovirus was packaged using Plat-A cells (#RV-102; Cell Biolabs). Fas-deficient RapoC2 Jurkat cells were previously described (Fu et al., 2016). RapoC2 Jurkat cells were transduced with retrovirus expressing Fas-BioID or BioID alone by spinfection at 1,500 × g at 32°C for 1 h with 8 µg/ml polybrene (#TR-1003-G; Sigma-Aldrich). Jurkat cells expressing Fas-BioID or BioID alone were enriched by FACS using EGFP expression. BioID-alone and Fas-BioID Jurkat cell lines were cultured in complete RPMI at 37°C (5% CO_2_), with 50 μM Biotin (#B4501; Sigma-Aldrich) added for the final 20 h of culture. In addition, Fas-BioID Jurkat cells were treated with 10 ng/ml FasL-LZ for the final 4 h of culture. A minimum of 30 × 10^6^ cells were harvested for BioID-only, Fas-BioID, and Fas-BioID+FasL-LZ conditions, with three replicates prepared per group. Cell pellets were snap-frozen prior to sample preparation. Cells were lysed using RIPA buffer (Sigma-Aldrich) with Halt inhibitor (Thermo) for 20 min on ice. Biotinylated proteins were pulled down using streptavidin agarose beads (Pierce) at 4°C overnight. Mass spectrometry and data analyses were performed by Poochon Biotech. The LC/MS/MS analysis of tryptic peptides for each sample was performed sequentially with a blank run between two sample runs using Thermo Fisher Scientific Orbitrap Exploris 240 Mass Spectrometer and Thermo Dionex UltiMate 3000 RSLCnano System (Poochon Biotech). Peptides from trypsin digestion were loaded onto a peptide trap cartridge at a flow rate of 5 μl/min. The trapped peptides were eluted onto a reversed-phase EASY-Spray Column PepMap RSLC, C18, 2 μM, 100A, 75 μm × 250 mm (Thermo Fisher Scientific) using a linear gradient of acetonitrile (3–36%) in 0.1% formic acid. The elution duration was 110 min at a flow rate of 0.3 μl/min. Eluted peptides from the EASY-Spray column were ionized and sprayed into the mass spectrometer, using Nano EASY-Spray Ion Source (Thermo Fisher Scientific) under the following settings: spray voltage, 1.6 kV; capillary temperature, 275°C. Raw data files were searched against human protein sequence database using Proteome Discoverer 2.4 software (Thermo Fisher Scientific) based on the SEQUEST algorithm. Carbamidomethylation (+57.021 Da) of cysteines was set as fixed modification, and oxidation/+15.995 Da (M) and deamidated/+0.984 Da (N, Q) were set as dynamic modifications. The minimum peptide length was specified to be five amino acids. The precursor mass tolerance was set to 15 ppm, whereas fragment mass tolerance was set to 0.05 Da. The maximum false peptide discovery rate was specified as 0.01.

#### Confocal and FLIM

Confocal microscopy and FLIM studies were performed using naïve and activated CD4 T cells from WT and *Pik3cd*^*E1020K/+*^ mice. Naïve CD4 T cells were isolated as previously described. For CD4 T cell activation, naïve CD4 T cells were cultured in the presence of APCs with αCD3 (1 μg/ml), αCD28 (3 μg/ml), and hIL-2 (50 U/ml). Following activation, CD4 T cells were isolated using Miltenyi CD4 isolation kits to remove APCs. Cells were then fixed with 4% PFA and stained with unconjugated αCD3ε (rabbit) and αFas (mouse) antibodies in PBS in suspension. Cells were subsequently washed and stained with α-rabbit-IgG Alexa Fluor 488 and α-mouse-IgG Alexa Fluor 555 secondary antibodies in PBS in suspension. Stained cells were prepared on slides, and coverslips were mounted using ProLong Gold antifade reagent (Thermo Fisher Scientific).

Confocal images were acquired on a DMi8 SP8 FALCON confocal microscope (Leica Microsystems). Images were acquired with fixed acquisition settings, and colocalization was determined using Imaris Image analysis software (Oxford Instruments, Bitplane). For FLIM, naïve and activated CD4 T cells were imaged on a Leica SP8 White Light Laser (WLL) FALCON inverted confocal microscope with a 63× oil immersion objective (Leica Microsystems). Donor lifetime measurements of Alexa Fluor 488 were measured by exciting at 488-nm wavelength by using a tunable WLL system tuned at 80-MHz frequency settings. Acquired fluorescent transients in the range of 1,000 or more photons per pixel were analyzed using LASX Single Molecule detection analysis software (LASX Single Molecule Detection software) to determine FL measurements. Mean FL and FRET efficiency calculations were observed and measured as described previously (Nabar et al., 2022).

#### Anti-CD3 crosslinking

For signaling studies with mouse CD4 T cells, naïve CD4 T cells were stimulated using APCs with anti-CD3ε (1 μg/ml) and anti-CD28 (3 μg/ml) in the presence of human IL-2 (50 IU/ml) for 72 h at 37°C, as previously described. Following culture, activated CD4 T cells were purified using a Miltenyi mouse CD4 T cell isolation kit. Purified activated CD4 T cells were resuspended in serum-free IMDM and rested at 37°C for 4 h. Cells were subsequently counted and coated with anti-CD3ε (1 μg/ml) in PBS for 15 min at 4°C. Cells were washed, resuspended in IMDM alone at 10 × 10^6^ cells/ml, and incubated at 37°C for 10 min. Samples were subsequently stimulated by adding prewarmed anti-Armenian hamster (5 μg/ml final) in IMDM to preincubated cells, and cells were fixed at desired time points in PFA (4% final, 20 min at room temperature). For samples that underwent anti-CD3 + FasL-LZ stimulation, FasL-LZ (25 ng/ml final) was mixed with anti-Armenian hamster and added to cells in tandem with the initiation of TCR stimulation. Cells were stained following the phospho-staining protocol outlined above.

#### ATACseq

1 × 10^4^ viable *in vitro* polarized Th2 cells were sorted into 500 μl of FACS buffer using an Aria Fusion Sorter (BD). Cells were pelleted and resuspended in 50uL of transposase mixture including 25 μl 2×TD buffer (Illumina), 2.5 μl TDE1 (Illumina), 0.5 μl 1% digitonin (Promega), and 22 μl water. Tagmentation was performed by incubation at 37°C for 30 min at 300 rpm. Following incubation, DNA was purified using a Qiagen MinElute kit, eluting samples in 10 μl. Purified tagemented DNA was PCR-amplified using previously described primers (Corces et al., 2016), with 12 cycles of amplification. Amplified libraries were purified using a Qiagen PCR cleanup kit and sequenced for 50 cycles (paired-end reads) on a NovaSeq 6000 (Illumina). ATACseq was done in three biological replicates per genotype. Reads were mapped to the mouse genome (mm10 assembly) using Bowtie 0.12.8. In all cases, redundant reads were removed using FastUniq, and customized Python scripts were used to calculate the fragment length of each pair of uniquely mapped paired-end reads. Reads whose fragment lengths were <175 bp were kept, and only one mapped read per unique genomic region was used to call peaks. Regions of open chromatin were identified by MACS (version 1.4.2) using a P value threshold of 1 × 10^−5^. Only regions called in three replicates were used for downstream analysis. Peak annotation and motif analysis were performed with the Hypergeometric Optimization of Motif EnRichment (HOMER) program version 4.11.1 using the following parameter settings: “annotatePeaks.pl peak_file mm10 -size 1000 -hist 40 -ghist” and “findMotifsGenome.pl peak_file mm10 motif_folder -size given -preparsedDir tmp 2 > out.” All peaks detected in WT and *Pik3cd*^*E1020K/+*^ Th2 were quantified for individual samples as follows: “annotatePeaks.pl peakfile mm10 -size given -raw -d ATACseq data,” and the differentially regulated peaks were called with a cutoff value of FDR <0.05 and log2FC >0.5 using the following command: “getDiffExpression.pl rawcount.txt -AvsA -DESeq2 -fdr 0.05 -log2fold 0.5 -simpleNorm.” Downstream analyses and graph generation were performed with R 4.1.1.

#### CTCF CUT&Tag

Naïve (liveTCRb^+^CD4^+^CD8^−^CD44^−^CD62L^+^CD25^−^), *in vitro* Th1-polarized (liveCD4^+^), and Th2-polarized (liveCD4^+^) CD4 T cells from WT and *Pik3cd*^*E1020K/+*^ mice were sorted using Aria Fusion Sorter (BD), as previously described and then subjected to CUT&Tag using the CUT&Tag/version 3 protocol (https://dx.doi.org/10.17504/protocols.io.bcuhiwt6) (Kaya-Okur et al., 2019) as indicated on isolated nuclei from naïve CD4 T cells and *in vitro* polarized Th1 and Th2 cells from WT and *Pik3cd*^*E1020K/+*^ mice. CUT&Tag utilized primary antibodies against CTCF (D31H2; Cell Signaling) and rabbit mAB IgG XP isotype control (DA1E; Cell Signaling). All primary antibodies were diluted 1:50 with the corresponding guinea pig anti-rabbit secondary antibody diluted 1:100 accordingly. pA-Tn5 was sourced preloaded (C01070001; Diagenode). Primary antibody incubation occurred for 1 h at room temperature with nutation and all other protocol steps as indicated in the native CUT&Tag protocol. Prepared libraries were normalized and pooled and prepared for sequencing via NovaSeq SP (Illumina) at the National Heart, Lung, and Blood Institute Genomics Core. Raw reads were processed using CutRunTools/20200629 (Zhu et al., 2019) with the mm10 mouse genome as reference. CTCF CUT&Tag signal data were RPKM-normalized using deepTools/3.5.5 with extendReads enabled. Peak calling for CTCF was performed using SEACR/version 1.3 with stringent settings and all fragment sizes incorporated. Peaks were further filtered by disassociation of peak summits belonging to the mm10 blacklist (CutRunTools) using bedtools/2.30.0 intersect. Peak signals were recalculated using deepTools computeMatrix ±200 bp and further filtered by at least twofold signal change over cell-type summed IgG signal. Differential CTCF peaks from either genotype were at least ± fourfold change in signal between respective genotypes. CTCF motif scoring was evaluated by HOMER/4.11.1 findMotifsGenome.pl with option -size 200. *Pik3cd*^*E1020K/+*^-induced and *Pik3cd*^*E1020K/+*^-repressed CTCF peaks were visualized using deepTools and mapped to nearest gene using HOMER annotatePeaks default settings.

#### Western blot

Naïve CD4 T cells were Th2-polarized as previously described, and live CD4 T cells were enriched using Miltenyi CD4 T cell isolation kits. For each condition, 1 × 10^6^ cells were pelleted and cells were lysed using 50 μl of 1% SDS in PBS (supplemented with protease inhibitor minitab [Sigma-Aldrich] and sodium orthovanadate) and 450 ml 1% Triton X in PBS (containing inhibitors). Cell lysates were sheared using a 25-gauge needle with a 1cc syringe. Lysates were centrifuged at 14,000 rpm for 15 min (4°C). Lysates were prepared with reducing sample buffer, and proteins were separated by SDS-PAGE and transferred to nitrocellulose. Membranes underwent blocking (TBS plus 5% BSA, 0.1% Tween-20) and were incubated with primary antibodies overnight (4°C). Subsequently, membranes were incubated with HRP-conjugated secondary antibodies for 60 min (room temperature). Signals were detected by chemiluminescence using Bio-Rad ChemiDoc Imaging System.

#### Statistical analysis

Data were analyzed using Prism version 9 (GraphPad software). All graphs show the mean ± SEM with *P < 0.05, **P < 0.01, ***P < 0.001, ****P < 0.0001. For unpaired comparisons, data were analyzed using unpaired *t* tests. For paired comparisons, data were analyzed using ratio paired *t* tests. For anti-CD3 crosslinking experiments, the area under the curve was calculated for individual signaling curves (baseline = 1) and peak areas were compared using ratio paired *t* tests.

### Material availability

No unique reagents were generated in this study. *Pik3cd*^*E1020K/+*^ mice are available from the lead author with a completed Materials Transfer Agreement.

### Online supplemental material

Fig. S1 contains supporting data for Fig. 1, including histological and scRNAseq analyses of HDM-treated animals. Fig. S2 contains supporting data for Fig. 2, including scRNAseq, flow cytometry, and Luminex analyses of HDM-treated WT and *Pik3cd*^*E1020K/+*^ mice. Fig. S3 contains supporting data for Figs. 3, 4, and 5, including flow cytometry and bulk RNAseq analyses of *in vitro* polarized Th2 cells. Fig. S4 contains supporting data for Fig. 6, including ATACseq, CUT&Tag, and western blot analyses. Fig. S5 contains supporting data for Figs. 7, 8, and 9, including analyses of Fas/FasL expression, signaling, and function in CD4 T cells. Table S1 contains cluster-specific gene expression for scRNAseq data shown in Fig. 1 G. Table S2 contains lists of Foxo1-activated and Foxo1-repressed genes used for GSEAs shown in Fig. 5, C, D, and F. Table S3 contains raw pathway enrichment analysis data for Fig. 6 G. Table S4 contains all raw Fas-BioID data corresponding to Fig. 8, D–G. Table S5 contains all antibodies used in this study. Table S6 contains all gRNA sequences.

## Supplementary Material

Table S1 contains cluster-specific gene expression for scRNAseq data shown in Fig. 1 G.

Table S2 contains lists of Foxo1-activated and Foxo1-repressed genes used for GSEAs shown in Fig. 5, C, D, and F.

Table S3 contains raw pathway enrichment analysis data for Fig. 6 G.

Table S4 contains all raw Fas-BioID data corresponding to Fig. 8, D–G.

Table S5 contains all antibodies used in this study.

Table S6 contains all gRNA sequences.

SourceData F6 is the source file for Fig. 6.

## Data Availability

This paper does not report original code. Sequence data have been deposited under GSE214871, GSE278564, GSE278337, and GSE277881 and will be available upon publication. Other data are included in supplemental materials. Any additional information required to reanalyze the data reported in this paper is available from the lead contact upon request.
