# Supplementary figures and images for: A PI3Kδ-Foxo1-FasL signaling amplification loop rewires CD4+ T cell signaling and differentiation
Source: J Exp Med. 2026 Feb 20;223(4):e20252154. doi: 10.1084/jem.20252154 (PMC12922663; doi:10.1084/jem.20252154)

Figure 6H

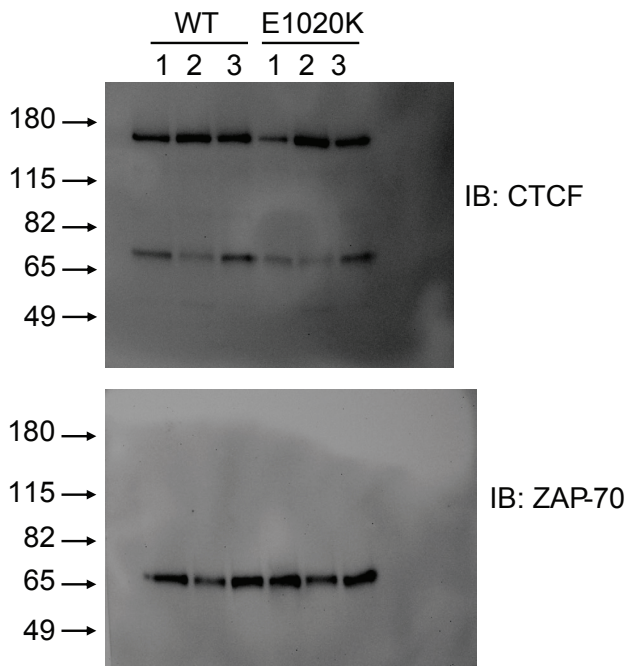

- 1) control
- 2) CAL-101
- 3) Rapamycin

Figure 6I

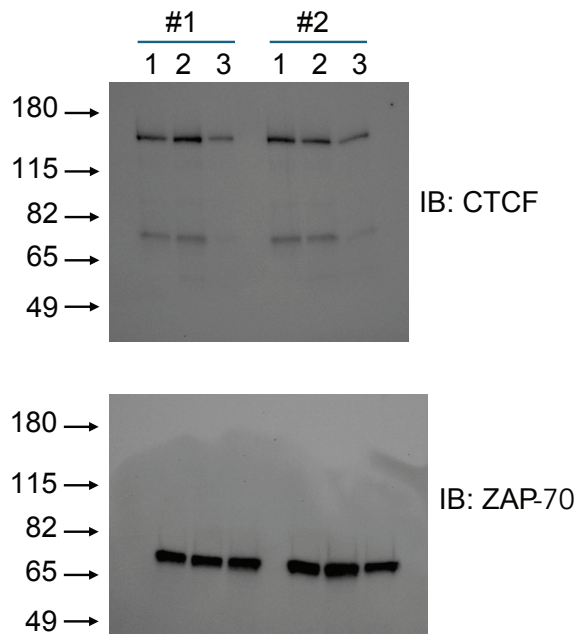

- 1) WT control
- 2) WT FoxO1 KO
- 3) E1020K

Supplement: SourceData F6 — is the source file for Fig. 6. [file jem_20252154_sourcedataf6.pdf]
